# Axitinib Has Antiangiogenic and Antitumorigenic Activity in Myxoid Liposarcoma

**DOI:** 10.1155/2016/3484673

**Published:** 2016-10-16

**Authors:** Lauren T. Kerr, Jacqueline F. Donoghue, Alexander L. Wilding, Terrance G. Johns

**Affiliations:** ^1^Centre for Cancer Research, Hudson Institute for Medical Research, 27-31 Wright Street, Clayton, VIC 3168, Australia; ^2^The Ritchie Centre, Hudson Institute for Medical Research, 27-31 Wright St, Clayton, VIC 3168, Australia; ^3^Monash University, Wellington Road, Clayton, VIC 3168, Australia

## Abstract

Myxoid liposarcoma is a rare form of soft-tissue sarcoma. Although most patients initially respond well to treatment, approximately 21% relapse, highlighting the need for alternative treatments. To identify novel treatment regimens and gain a better understanding of myxoid liposarcoma tumor biology, we screened various candidate and approved targeted therapeutics and chemotherapeutics against myxoid liposarcoma cell lines. Therapeutics that target angiogenesis showed antitumor activity. The small molecule inhibitor axitinib, which targets angiogenesis by inhibiting the VEGFR and PDGFR families and c-Kit, inhibited cell cycle progression and induced apoptosis* in vitro*, as well as having significant antitumor activity against MLS 1765 myxoid liposarcoma xenografts in mice. Axitinib also displayed synergistic antitumor activity* in vitro* when combined with the potassium channel ionophore salinomycin or the BH3 mimetic ABT-737. Another angiogenesis-targeting therapeutic, 4EGI-1, which targets the oncoprotein eIF4E, significantly decreased angiogenic ligand expression by myxoid liposarcoma cells and reduced tumor cell growth. To verify this oncogenic addiction to angiogenic pathways, we utilized VEGFR-derived ligand traps and found that autocrine VEGFR signaling was crucial to myxoid liposarcoma cell survival. Overall, these findings suggest that autocrine angiogenic signaling through the VEGFR family is critical to myxoid liposarcoma cell survival and that further study of axitinib as a potential anticancer therapy is warranted.

## 1. Introduction

Myxoid liposarcoma is a rare malignant tumor that arises from mesenchymal tissue, and tumor grade is based on the percentage of round cell morphology. Approximately, two-thirds of MLS tumors arise in the musculature of the thigh, and the remaining one-third occur in deep fatty tissue. On rare occasions, MLS can be found in the retroperitoneum or subcutaneously [1]. About 600 people are diagnosed with myxoid liposarcoma each year in the United States [2]. Current treatment involves surgical resection including clear margins, with 74% of patients undergoing radiation therapy as well. In 40% of patients, chemotherapy such as doxorubicin or ifosfamide is also included because of the presence of round cells. MLS with round cells are considered highly metastatic with more than 21% of patients developing metastases or local recurrence [3]. Therefore, an improved understanding of the tumor biology and investigations into new treatment options are warranted.

Myxoid liposarcoma is a unique cancer as >95% of tumors contain a reciprocal chromosomal translocation, t(12;16)(q13;p11), which produces the chimeric fusion protein FUS-CHOP (also known as FUS-DDIT3) [4, 5]. FUS-CHOP drives tumorigenesis in myxoid liposarcoma by interfering with the expression of transcription factors (including PPAR*ϒ*1, PPAR*ϒ*2, C/EBP*α*, C/EBP*β*, and C/EBP*δ*) that regulate the differentiation of adipocyte precursor cells. This alteration drives the preadipocyte cells into a continuous cycle of proliferation without differentiation, leading to malignancy [6]. Moreover, transgenic mice that ubiquitously express FUS-CHOP develop myxoid liposarcoma-like tumors at adipose tissue sites. This finding suggests that FUS-CHOP causes myxoid liposarcoma and is sufficient to drive transformation [7]. The expression of the oncogene FUS-CHOP is believed to be involved in myxoid liposarcoma tumor initiation [8]. Approximately 50% of myxoid liposarcoma cells express nuclear FUS-CHOP [9], and cells that are negative for CHOP express high levels of proliferation markers [10]. This inverse relationship between proliferation and FUS-CHOP expression results in a population of senescent cells [10]. Senescence results in apoptosis and necrosis, which is common in many tumors. As a result, tissue hypoxia ensues, along with inflammation, contributing to the oncogenic transformation of the microenvironment through cytokine release and angiogenesis. This population of cells in myxoid liposarcoma may therefore be reflective of oncogenic mechanisms, adding further to the complexity of these tumors. Thus, targeting FUS-CHOP or its downstream mediators may be therapeutically efficacious.

In order to identify novel treatment strategies and characterize the tumor biology of myxoid liposarcoma, we employed the patient-derived MLS-402-91 and MLS-1765-92 cell lines first described by Aman et al. in 1992 [11]. In a recent study, 18% of myxoid/round cell liposarcomas were shown to express activating* PI3KCA* mutations [12], whereas 100% of myxoid liposarcoma samples (17/17) expressed wild-type* PI3KCA* and 67% of round cell liposarcomas (4/6) expressed* PI3KCA* mutations [13]. This indicated that* PI3KCA* mutation status can be used to partition the two liposarcoma groups into myxoid and round cell types. Furthermore, the poor survival response of patients with these tumors was related to the round cell component. The MLS-402-91 and MLS-1765-92 cell lines used in our study express wild-type* PI3KCA* [13] and therefore reflect the genomic landscape of the myxoid liposarcoma population. These sarcoma cell lines were therefore used in this study to assess the antiproliferative and antitumorigenic activity of a panel of approved and candidate targeted therapeutics and chemotherapeutics* in vitro* and* in vivo*.

## 2. Materials and Methods

### 2.1. Panel of Drugs and Drug Candidates

The following 43 reagents were used in this study: AMG 102 and panitumumab (Amgen); cercosporamide (BioAustralis); AKT inhibitor V (Calbiochem); AS-252424, bisindolylmaleimide, CGP 57380 and imatinib (Cayman Chemical); CTX ILK inhibitor (CRC); Avastin (bevacizumab) (Genentech/Roche); 4EGI-1, ABT-737, ABT-737 enantiomer, pazopanib, and retinoic acid (Santa Cruz Biotechnology); erlotinib, lapatinib, sorafenib, and temsirolimus (Scientifix); bortezomib and CYT387 (Selleck Chemicals); AKT-I-1/2, axitinib, bicalutamide, cilostazol, cyclopamine, DAPT, dasatinib, docetaxel, doxorubicin, floxuridine, fluorouracil, goserelin, ifosfamide, PD98059, ribavirin, salinomycin, SU11274, sunitinib, Tamoxifen, and vinblastine (Sigma); NSC 7908 (Tocris); and temozolomide (Wyeth).

### 2.2. Cell Culture

Two SV40-transfected, patient-derived myxoid liposarcoma cell lines were used: MLS-402-91 (MLS 402) and MLS-1765-92 (MLS 1765). Both were generated by Aman et al. (Lundberg Laboratory for Cancer Research, University of Gothenburg, Sweden) [11]. SW872, a liposarcoma cell line without FUS-CHOP, was obtained from the ATCC. The myxoid liposarcoma cell lines were maintained in RPMI medium containing 10% FCS, GlutaMAX, and penicillin/streptomycin (Invitrogen). HUVECs were generously donated by P. Rogers (Melbourne University, Melbourne, Australia) and maintained in EGM-2 BulletKit medium (Lonza). U87 cells were sourced from the ATCC (Manassas) and maintained in DMEM/F12 medium (Invitrogen) containing 5% FCS, GlutaMAX and penicillin/streptomycin.

### 2.3. Antibodies, Immunoprecipitation, and Western Blotting

Briefly, vascular endothelial growth factor receptor 3 (VEGFR3) was immunoprecipitated (IP) from myxoid liposarcoma lysates using a rabbit anti-human VEGFR3 polyclonal antibody (C-20, 1 : 20; Santa Cruz Biotechnology) and protein A/G agarose beads (Santa Cruz Biotechnology). The mixture was incubated for 18 h at 4°C with rotation. The beads were washed and boiled for 5 min at 95°C in reducing buffer to elute the protein. Western blotting was carried out as previously described [14]. Total VEGFR3 was detected using a rabbit anti-human VEGFR3 polyclonal antibody (C-20, 1 : 200); phospho-VEGFR3 was detected using a mouse anti-human pan-phospho-tyrosine monoclonal antibody (135900, 1 : 2,000; Invitrogen). The following antibodies were also used for western blotting: a mouse anti-human CHOP monoclonal antibody (L63F7, 1 : 1,000; Cell Signaling Technology) and a rabbit anti-human eukaryotic translation initiation factor 4E (eIF4E) monoclonal antibody (C46H6, 1 : 1,000; Cell Signaling Technology). Species-specific Alexa Fluor 680 IgG (Invitrogen) secondary antibodies were used for both immunoprecipitation and western blotting.

### 2.4. Cell Proliferation (MTS) Assay

An MTS dye uptake assay was performed to measure cell proliferation. MTS reagent (20 *μ*L) was added to 96-well plates (cell titer 96 aqueous nonradioactive cell proliferation [Promega]), and plates were incubated for 2.5 h at 37°C in 5% CO_2_. The absorbance was measured at 490 nm using a FLUOstar Optima spectrophotometer and software (version 2.2OR2).

### 2.5. siRNA Knockdown

Myxoid liposarcoma cells were seeded on plates and incubated for 18 hours at 37°C in 5% CO_2_. Then siRNAs targeting CHOP or eIF4E or control scramble siRNAs were added as outlined in the manufacturer's protocols (Dharmacon ON-Targetplus SMARTpool, Thermo Fisher Scientific). The plates were incubated for 5 days at 37°C in 5% CO_2_ and then an MTS assay or western blotting was performed.

### 2.6. Tube Formation Assay

Matrigel Growth Factor Reduced Basement Membrane Matrix (22 mg/mL [BD Biosciences]) was diluted to 8.8 mg/mL in cold, sterile PBS, and 100 *μ*L was transferred into each well of a chilled 96-well plate. The plate was incubated for 2 h at 37°C to set the Matrigel. HUVEC cells were washed with PBS, lifted from the flask, and washed again with serum-free basal EGM-2 medium (Lonza). Then, 10,000 cells were added per well together with control medium or medium from MLS 402 cells pretreated with drug. The negative control medium was basal EGM-2 medium (serum-free), and the positive control medium was EGM-2 medium supplemented with growth factors and 2% FCS that were supplied with the medium. The plate was incubated at 37°C in 5% CO_2_, and images were recorded at 1 h intervals for 12 h by a ProgRes MF cool camera attached to an Axiovert 40 CFL microscope, using ProgRes Mac Capture software (version 2.8.3). Tube lengths were measured using ImageJ (version 1.47d).

### 2.7. Drug Screen

Myxoid liposarcoma cells were seeded into 96-well plates at 1,000–2,000 cells per well and incubated for 18 h at 37°C in 5% CO_2_. The drugs listed above were then added to the plate in triplicate at a final concentration of 10 *μ*M, except for bevacizumab (10 mg/mL), and the plates were incubated at 37°C in 5% CO_2_ for 5 days. An MTS assay was performed to calculate cell viability. Isobolograms were calculated using the Loewe additivity method [15], while the Chou and Combination Index (CI) plots were generated using the Chou and Talalay method [16].

### 2.8. RT-qPCR

Briefly, myxoid liposarcoma cells were lifted from the flask and washed with PBS, and total RNA was extracted using the RNeasy Mini Kit (QIAGEN) as per the manufacturer's instructions. The RNA was DNase treated using an RQ1 RNase-Free DNase kit (Promega), as outlined in the manufacturer's protocol, and then converted into cDNA using a SuperScript III First-Strand Synthesis System kit (Invitrogen) as per the manufacturer's instructions. Primers and probes specific for VEGFR1, VEGFR2, VEGFR3, VEGFA, VEGFB, and cyclin D1 (with a FAM probe and a nonfluorescent quencher [Applied Biosystems]) were used. cDNA (2.5 *μ*L) was added to the primer/probe set and TaqMan Gene Expression Master Mix (Applied Biosystems), following the manufacturer's protocol. PCR was carried out on a 7900 fast real-time PCR thermocycler (Applied Biosystems) under standard cycling conditions. The data were analyzed using SDS 2.3 software (Applied Biosystems). The values were normalized to the housekeeping genes* H6PD* or* GAPDH*. Relative quantification was determined using the 2^(control  –  sample)^ method [17].

### 2.9. Ligand Traps

Myxoid liposarcoma cells were seeded at a density of 1,000–2,000 cells per well on 96-well plates and incubated at 37°C in 5% CO_2_ for 18 h. Then ligand traps were added at 2.0 *μ*g/100 *μ*L: recombinant human VEGFR1 (FLT-1)-Fc chimera (R&D Systems), recombinant mouse VEGFR3 (FLT-4)-Fc chimera (R&D Systems), both of these ligand traps, or vehicle control. The plates were incubated at 37°C in 5% CO_2_ for 3 days, and then an MTS assay was performed.

### 2.10. Bio-Plex and MILLIPLEX Assays

Both Bio-Plex (Bio-Rad) and MILLIPLEX (Millipore) kits were used to quantify the total levels and/or the levels of phosphorylated proteins. Briefly, myxoid liposarcoma cells were seeded at a density of 200,000 cells per well in a 6-well plate and incubated for 18 h at 37°C in 5% CO_2_. Cells were then treated for 2 h with axitinib (IC_50_) or vehicle control, washed with PBS, and lyzed using the buffer provided. The manufacturer's instructions were then followed. The Bio-Rad Bio-Plex 200 System was used to measure the plates, and the data were analyzed using the software Bio-Plex Manager 5.0.

### 2.11. Cell Cycle Analysis and Annexin V Staining

Myxoid liposarcoma cells (1 × 10^6^) were seeded in 25 cm^2^ flasks, incubated for 18 h at 37°C in 5% CO_2_, and then treated with axitinib (IC_50_) or vehicle control for 18 h. The cell monolayers were then washed, and the cells were lifted from the wells and counted.

For the cell cycle assay, 1 × 10^6^ cells were resuspended in 1 mL PBS with 25 *μ*L propidium iodide (100 *μ*g/mL, Sigma) and then analyzed by using a Becton Dickinson FACS Canto II. Fluorescence signals for DNA-propidium iodide were detected using a 585/42 nm bandpass filter. The distribution of cells containing DNA characteristic of the G1, S, and G2/M cell cycle phases was determined using FlowJo software (version 7.5.5).

The annexin V/7AAD assay was performed using a PE Annexin V Apoptosis Detection Kit 1 (Becton Dickinson), as per the manufacturer's instructions. Fluorescent signals for annexin V-PE were detected using a 585/42 nm bandpass filter. The data were analyzed using FlowJo software.

### 2.12. *In Vivo* Mouse Study

Our research was approved by Monash Medical Centre Animal Ethics Committee A and conducted in accordance with Monash University and NHMRC guidelines. Mice were kept in pathogen-free conditions with a 12 h light:dark cycle at 23° ± 2°C. Mice were provided with food and water* ad libitum*. The acclimatization period was 2 weeks. Nonobese diabetic-severe combined immunodeficient (NOD-SCID) mice were sourced from Monash Animal Services (Melbourne, Australia).

Into 6–8-week-old female NOD-SCID mice, 7 × 10^6^ MLS 1765 cells were injected subcutaneously into both flanks. The proportion of tumors that grew was small; therefore, for the* in vivo* drug treatment experiments, we transplanted growing tumor into the flanks of new mice as follows: when the tumors grown from cells reached 1,000 mm^3^, they were excised and disassociated, and tumor pieces totaling 100 mm^3^ were transplanted into the flanks of new donor NOD-SCID mice. This procedure had the advantage that almost all tumors grew and that tumors were not undergoing growth adaptation during drug treatment. Tumors that had been serially transplanted five times (P5) (see Supplementary Figure  S10 in Supplementary Material available online at http://dx.doi.org/10.1155/2016/3484673) were used for therapeutic studies.

When tumors were approximately 200 mm^3^, mice were randomized into control and treatment groups, and treatment began. This tumor size was chosen to enable sufficient duration of drug treatment before tumors reached the maximum ethically permitted size, 1,000 mm^3^. Mice were injected every second day with 30 mg/kg axitinib or vehicle control for 12 days. Tumors were measured periodically using digital calipers, and tumor volumes were calculated using the formula (length × width^2^)/2. Two days after the final injection, mice were culled, and the tumors were excised, weighed, and photographed. Mice were monitored daily, and if tumors grew to more than 1,000 mm^3^, mice were humanly euthanized.

### 2.13. Statistical Analysis

Data were analyzed using GraphPad Prism (version 6). Student's *t*-test was used for pairwise analysis. Statistical significance was set at *p* ≤ 0.05.

See Supplementary Methods for further detail on dose-response curves and combination drug trials.

## 3. Results

### 3.1. 4EGI-1 and Axitinib Have Antiproliferative Activity against Myxoid Liposarcoma Cells

**Figure 1:**
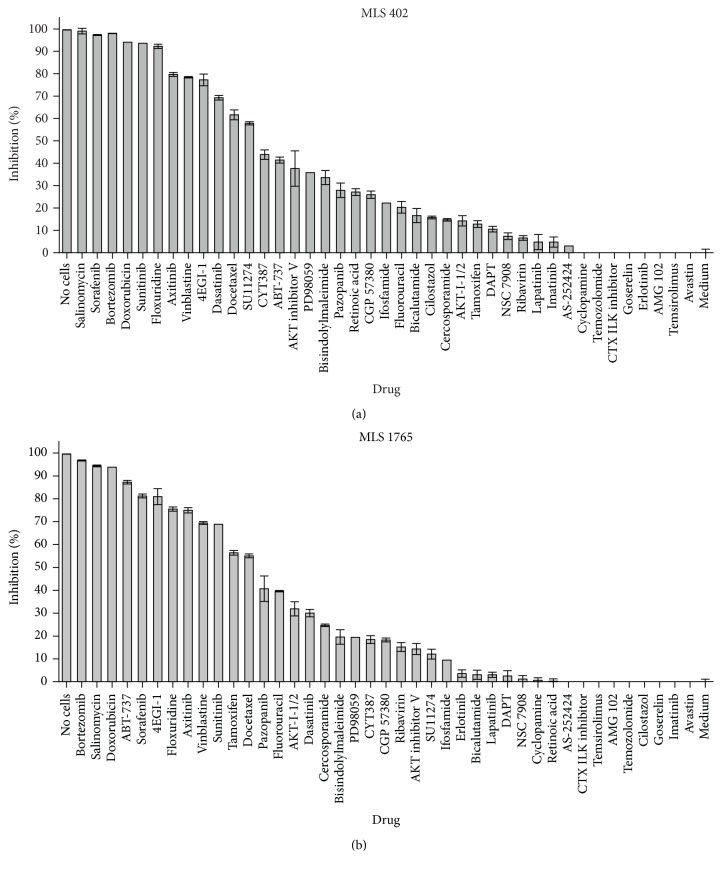
The effect of various drugs on myxoid liposarcoma cell proliferation. MLS 402 and MLS 1765 cells were treated with 10 *μ*M drug (10 mg/mL bevacizumab) for 5 days. Following MTS dye uptake assay, cell viability was determined. Various agents inhibited the proliferation of MLS 402 (a) and MLS 1765 (b). Tests were performed on three technical replicates. The data are presented as the mean ± SEM and are expressed as the percentage inhibition compared with vehicle-treated cells.

To identify drugs with antiproliferative activity, we screened 43 drugs for their* in vitro* antiproliferative activity against two myxoid liposarcoma patient-derived cell lines, MLS 402 and MLS 1765, which have both been confirmed to express FUS-CHOP [18]. The panel included both chemotherapeutics and targeted therapeutics and was selected on the basis of targeting cancer-specific proteins. Each drug was tested at 10 *μ*M, the highest dose with therapeutic relevance. The proliferation of myxoid liposarcoma cells was inhibited, as determined by the MTS assay, in the presence of agents that induced apoptosis (ABT-737 [MLS 402, 14.1 *μ*M; MLS 1765, 12.8 *μ*M] and salinomycin [MLS 402, 1.3 *μ*M; MLS 1765, 1.3 *μ*M]) or targeted receptor tyrosine kinase (RTK) inhibitors (axitinib [MLS 402, 1.2 *μ*M; MLS 1765, 3.2 *μ*M], dasatinib [MLS 402, 1.6 *μ*M; MLS 1765, 4.0 *μ*M], sorafenib [MLS 402, 10.4 *μ*M; MLS 1765, 9.9 *μ*M], and sunitinib [MLS 402, 3.8 *μ*M; MLS 1765, 1.7 *μ*M]), as well as the proteasome inhibitor bortezomib [MLS 402, 0.03 *μ*M; MLS 1765, 0.06 *μ*M] and the eIF4E inhibitor 4EGI-1 [MLS 402, 8.2 *μ*M; MLS 1765, 4.8 *μ*M] (Figures 1(a) and 1(b)). Myxoid liposarcoma cells were also highly sensitive to the chemotherapeutics doxorubicin and floxuridine.

Next, we assessed the efficacy of the agents with the highest antiproliferative activity (those that reduced cell viability by 70% or more in the screening assay), by measuring the antiproliferative activity of a drug dilution series (Supplementary Figure S1). For the targeted therapeutics, the order of antiproliferative activity (highest to lowest), as determined from the half-maximal inhibitory concentration (IC_50_) values (Supplementary Table 1), was CYT-387, salinomycin, axitinib, dasatinib, sunitinib, and 4EGI-1.

To identify more potent treatment strategies than those with single agents, we combined pairs of drugs. Drug combinations were selected based on high sensitivity (i.e., a low IC_50_) with a preference for targeted therapies and rational combinations (e.g., RTK inhibitors and apoptosis inducers) (Supplementary Table 2 and Supplementary Figures S2–S8). We examined the effect of the drug pairs on cell growth, both alone and in combination, by using an MTS proliferation assay. Several pairs, particularly combinations containing the proapoptotic drug salinomycin, demonstrated enhanced antiproliferative activity when combined. The combination of axitinib and salinomycin had synergistic activity against MLS 1765 and additive activity against MLS 402 (Supplementary Figure S2). The ABT-737 and salinomycin combination had synergistic activity against both myxoid liposarcoma cell lines (Supplementary Figure S5). Dasatinib plus salinomycin (Supplementary Figure S6) and ABT-737 plus axitinib (Supplementary Figure S7) had synergistic activity against MLS 1765. The 4EGI-1 and salinomycin combination had synergistic activity against MLS 402 and additive activity against MLS 1765 (Supplementary Figure S4). In contrast, the combination of axitinib and 4EGI-1 was antagonistic for both cell lines (Supplementary Figure S3), and when either was combined with doxorubicin, no enhanced cell death was observed (Supplementary Figure S8). These results indicate that combination drug therapy involving proapoptotic agents and targeted therapeutics may be highly efficacious against myxoid liposarcoma.

### 3.2. FUS-CHOP and eIF4E Are Critical for Myxoid Liposarcoma Cell Survival

We selected two of the agents from the panel based on their high antiproliferative activity and known ability to target myxoid liposarcoma-specific proteins—4EGI-1 and axitinib—and characterized the importance of their targets in myxoid liposarcoma. We also characterized the importance of FUS-CHOP, given the widespread presence of this fusion protein in myxoid liposarcoma. This target was not examined in the screen because there are no candidates or approved therapeutics that target FUS-CHOP.

**Figure 2:**
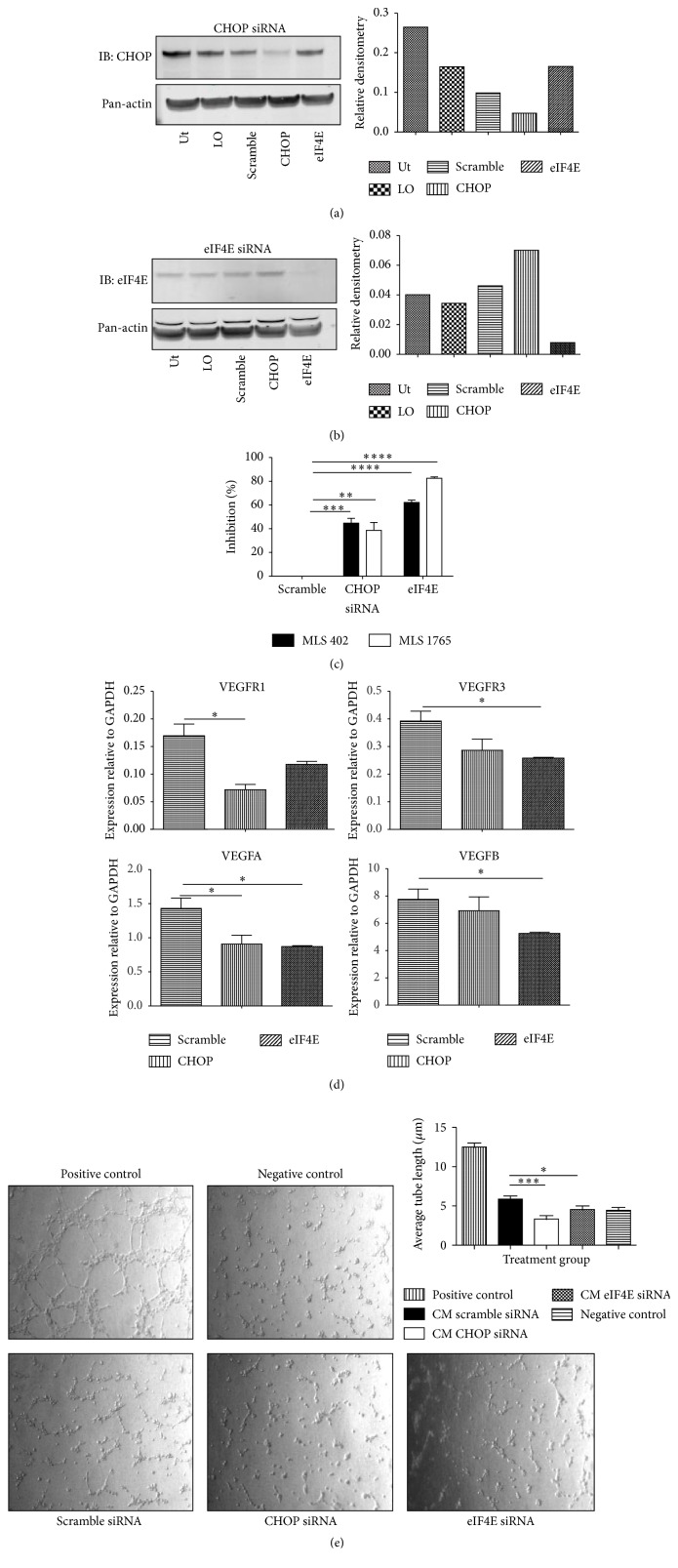
FUS-CHOP and eIF4E are critical for myxoid liposarcoma survival and promote the angiogenic properties of myxoid liposarcoma. MLS 1765 cells were treated with CHOP-directed (a), eIF4E-directed (b), or scramble siRNA and the percentage of protein reduction was determined by densitometry. MLS 402 and MLS 1765 cells were treated with CHOP-directed, eIF4E-directed, or scramble siRNA for 5 days and cell viability determined by MTS uptake (c). Three independent experiments were performed, and the data are presented as the mean + SEM. MLS 1765 cells treated with CHOP-directed, eIF4E-directed, or scramble siRNA for 5 days, and then RT-qPCR was performed to quantify the expression of VEGF ligands and receptors. Data are presented relative to the housekeeping gene GAPDH; three independent experiments were performed; and the data are presented as the mean + SEM (d). HUVECs were suspended in Matrigel, and then conditioned medium from MLS 402 cells pretreated with CHOP-directed, eIF4E-directed, or scramble siRNA was applied to the HUVECs. Images were acquired at hourly intervals, and the figure displays representative images taken at 8 h (left). Tube lengths were measured using ImageJ (version 1.47d). Three independent experiments were performed, and the data are presented as the mean + SEM (e). CM, conditioned medium; IB, immunoblotting; LO, Lipofectamine-only treated cells; Ut, untreated MLS cells. A paired, two-tailed *t*-test was performed. ^*∗*^ *p* < 0.05; ^*∗∗*^ *p* < 0.01; ^*∗∗∗*^ *p* < 0.001; ^*∗∗∗∗*^ *p* < 0.0001.

The success of the eIF4E inhibitor 4EGI-1 in our initial screen pointed to a role for this oncoprotein in myxoid liposarcoma. This is supported by a previous report showing that eIF4E is overexpressed in myxoid liposarcoma and may by critical to tumor development [6]. To investigate the importance of FUS-CHOP and eIF4E expression in myxoid liposarcoma, we performed siRNA knockdown of CHOP (using a CHOP-directed siRNA, which also targets FUS-CHOP) or eIF4E in the myxoid liposarcoma cell lines. The knockdown of FUS-CHOP was specific for FUS-CHOP and not wild-type CHOP, as determined by performing a western blot with a series of drugs known to induce wild-type CHOP expression (Supplementary Figure S9(A)). Moreover, wild-type CHOP was not present in myxoid liposarcoma cell lines (Supplementary Figure S9(C)). Therefore, the siRNAs were specific and functioned as expected. Treatment with either siRNA resulted in a marked reduction in protein expression compared with treatment with Lipofectamine only: the amount of FUS-CHOP protein was reduced by 60% in each myxoid liposarcoma cell line (Figure 2(a) and Supplementary Figure S9(A)), and the amount of eIF4E protein was reduced by 75% in MLS 402 and 82% in MLS 1765 (Figure 2(b) and Supplementary Figure S9(B)) (CHOP: MLS 402, *p* = 0.004, and MLS 1765, *p* = 0.045; eIF4E: MLS 402 and MLS 1765, *p* < 0.0001).

We then investigated the influence of siRNA-mediated knockdown of CHOP or eiF4E on cell survival and proliferation by using an MTS proliferation cells treated with CHOP-directed siRNA proliferated significantly less than cells treated with scramble control siRNA, with a 45% reduction for MLS 402 cells and a 39% reduction for MLS 1765 cells (Figure 2(c)). Similarly, eIF4E-directed siRNA significantly reduced cell proliferation, by 62% for MLS 402 cells and 83% for MLS 1765 cells (Figure 2(c)). Therefore, both eIF4E and FUS-CHOP are critical to myxoid liposarcoma cell proliferation and survival.

### 3.3. FUS-CHOP and eIF4E Promote Angiogenic Properties

In addition to tumor cell proliferation, VEGFR signaling, which promotes angiogenesis, has been implicated as a driver of myxoid liposarcoma and other sarcomas [19–21]. Specifically, VEGFA is universally detected in human myxoid liposarcoma tumors [22], and FUS-CHOP has been shown to upregulate VEGFR1 when expressed in HT1080 human fibrosarcoma cells [23]. Consistent with these findings, our drug screen identified that the VEGFR inhibitors axitinib, sorafenib, and sunitinib are potent inhibitors of myxoid liposarcoma cell growth. Similarly, eIF4E has also been shown to elevate angiogenic factors [24]. To examine a potential association between FUS-CHOP and eIF4E expression and the angiogenic activity of myxoid liposarcoma cells, we examined the effect of siRNA knockdown on the expression and activity of proangiogenic factors.

Knockdown of FUS-CHOP with CHOP-directed siRNA significantly reduced the expression of VEGFR1 (*p* = 0.014) and its ligand, VEGFA (*p* = 0.05), compared with scramble siRNA in MLS 1765 cells (Figure 2(d)). In addition, medium derived from MLS 402 cells treated with CHOP-directed siRNA significantly reduced endothelial cell tube formation compared to positive control medium (*p* = 0.001) (Figure 2(e)), further highlighting the angiogenesis-promoting properties of FUS-CHOP.

Similarly, eIF4E knockdown significantly reduced the expression of VEGFR3 (*p* = 0.02) and the ligands for VEGFR1, VEGFA (*p* = 0.02), and VEGFB (*p* = 0.03) compared with scramble siRNA in MLS 1765 cells (Figure 2(d)). Conditioned medium from MLS 402 cells pretreated with eIF4E-directed siRNA also significantly reduced endothelial cell tube formation compared with conditioned medium from MLS 402 cells pretreated with scramble siRNA (*p* = 0.012) (Figure 2(e)).

**Figure 3:**
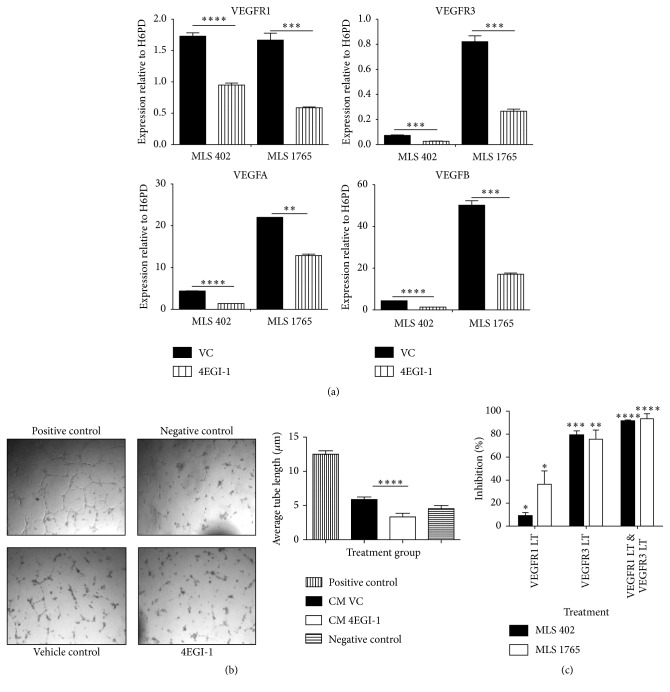
eIF4E promotes angiogenic properties. MLS 402 and MLS 1765 cells were treated with IC_50_ 4EGI-1 (MLS 402, 8.2 *μ*M; MLS 1765, 4.8 *μ*M) or vehicle control (VC) overnight, and then RT-qPCR was performed to measure the expression of VEGF ligands and receptors. The assay was performed three times. The data shown are from three independent experiments presented as mean + SEM relative to the housekeeping gene H6PD (a). HUVECs were suspended in Matrigel, and then conditioned medium from MLS 402 cells pretreated with IC_50_ 4EGI-1 or vehicle control overnight was applied. Images were acquired at hourly intervals, and the figure displays representative images taken at 8 h. Tube length was measured using ImageJ (version 1.47d) (b). CM, conditioned medium; IB, immunoblotting; LO, Lipofectamine-only treated cells; Ut, untreated MLS cells. MLS 402 and MLS 1765 cells were plated overnight and then treated with 20 *μ*g/mL VEGFR1 ligand trap (LT), VEGFR3 ligand trap, both ligand traps, or vehicle control for 3 days. Then an MTS dye uptake assay was performed to measure cell viability, and the effect on cell proliferation was calculated (c). Technical duplicates and biological triplicates were tested. A paired, two-tailed *t*-test was performed. The data are presented as mean + SEM. ^*∗*^ *p* < 0.05; ^*∗∗*^ *p* < 0.01; ^*∗∗∗*^ *p* < 0.001; ^*∗∗∗∗*^ *p* < 0.0001.

To confirm these findings, we pharmaceutically inhibited eIF4E by using 4EGI-1. MLS 1765 cells and MLS 402 cells had significantly reduced expression of VEGFR1 (MLS 402, *p* < 0.0001; MLS 1765, *p* = 0.0006), VEGFR3 (MLS 402, *p* = 0.0006; MLS 1765, *p* = 0.0004), VEGFA (MLS 402, *p* < 0.0001; MLS 1765, *p* = 0.001), and VEGFB (MLS 402, *p* < 0.0001; MLS 1765, *p* = 0.0003) following 4EGI-1 treatment, compared with the vehicle-control-treated cells (Figure 3(a)). The other VEGFR receptor, VEGFR2, is expressed at only trace levels in myxoid liposarcoma cell lines (Supplementary Figure  9(D)); thus, inhibition of VEGFR2 is unlikely to affect myxoid liposarcoma cell lines. The loss of VEGF ligands and receptor expression after pharmaceutical inhibition with 4EGI-1 confirmed that eIF4E promotes some angiogenic properties of myxoid liposarcoma cell lines. This finding was further verified by a significant reduction in angiogenic ligands in conditioned medium from cells pretreated with 4EGI-1, resulting in a significant reduction in endothelial cell tube formation compared to vehicle control (*p* = 0.012) (Figure 3(b)). Thus, both eIF4E and FUS-CHOP contribute to the angiogenesis observed in myxoid liposarcoma cells through the regulation of angiogenic receptors and ligands.

### 3.4. VEGFR Signaling Is Required for Cell Proliferation

To establish the dependence of myxoid liposarcoma cell lines on the autocrine activity of angiogenic receptors and their ligands, we measured cell proliferation changes in response to VEGFR1 and VEGFR3 ligand traps, which mimic the respective receptors and sequester their ligands, thereby preventing receptor activity. The ligand traps significantly inhibited cell proliferation both alone [MLS 402 (VEGFR1 by 9%, *p* = 0.035; VEGFR3 by 79%, *p* = 0.0006) and MLS 1765 (VEGFR1 by 31%, *p* = 0.042; VEGFR3 by 76%, *p* = 0.0015)] and in combination [MLS 402 (VEGFR1 + VEGFR3 by 91%, *p* = 0.0004) and MLS 1765 (VEGFR1 + VEGFR3 by 94%, *p* = 0.0074)] compared with vehicle treatment (Figure 3(c)). These data therefore indicate that VEGFR1 and VEGFR3 promote myxoid liposarcoma cell proliferation, with these cells having a strong dependence on VEGFR3 signaling.

### 3.5. Axitinib Inhibits the Phosphorylation of Angiogenic Receptors

Given the above results, we decided to characterize the antitumor effects of the antiangiogenic drug axitinib, which had high antiproliferative activity, in detail. Given the importance of VEGFRs in both angiogenesis and tumor cell proliferation, we investigated axitinib's effects on the activation of angiogenic receptors, activation of signal transduction molecules, expression of angiogenic molecules, formation of endothelial tubes, progression of the cell cycle, apoptosis, and growth of tumor xenografts.

**Figure 4:**
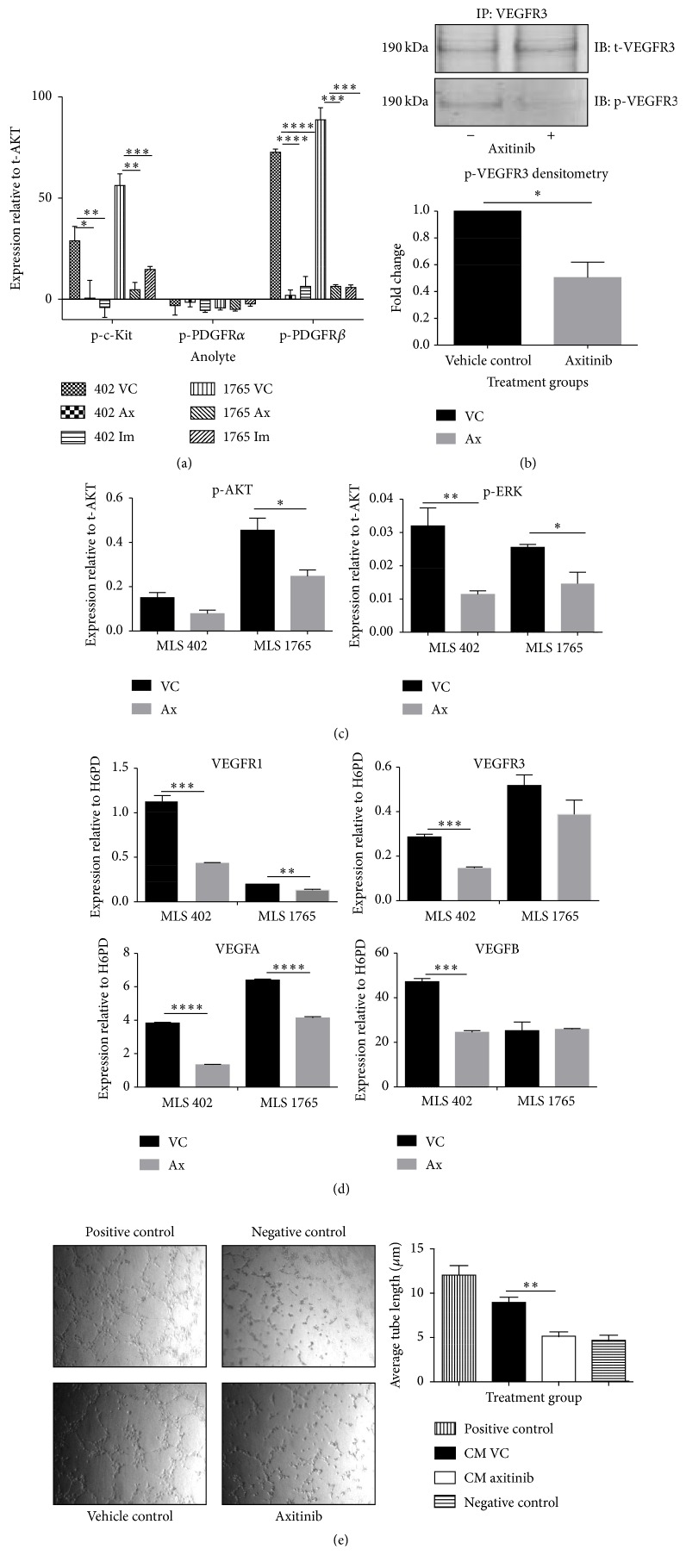
Characterization of axitinib. MLS 402 and MLS 1765 cells were treated with IC_50_ axitinib (MLS 402, 1.2 *μ*M; MLS 1765, 3.2 *μ*M), imatinib (10 *μ*M), or vehicle control for 2 h, lyzed, and analyzed for anolytes targeting the phosphorylation site of PDGFR*α*, PDGFR*β*, and c-Kit. The experiment was performed 3 times, and the data are presented as mean + SEM. t-AKT, total AKT (a). MLS 1765 cells were treated overnight with IC_50_ axitinib or vehicle control and immunoprecipitated for total VEGFR3 (t-VEGFR3) and phospho-VEGFR3 (p-VEGFR3). Densitometry of total VEGFR3 expression and phospho-VEGFR3 expression following axitinib treatment (b). MLS 402 and MLS 1765 cells were treated with IC_50_ axitinib or vehicle control for 2 h, and then the cells were lyzed and analyzed for phospho-AKT and phospho-ERK1/2 by Bio-Plex. Three technical and biological replicates were tested. The data are presented as the mean + SEM (c). MLS 402 and MLS 1765 cells were treated overnight with IC_50_ axitinib or vehicle control, and then cells were analyzed by RT-qPCR for the expression of VEGFR1, VEGFR3, VEGFA, and VEGFB. The experiment was performed three times, and the data are presented as the mean + SEM (d). HUVECs were suspended in Matrigel and then were treated with conditioned medium from cells that had been pretreated with IC_50_ axitinib or vehicle control. Images were acquired at hourly intervals, and the figure displays representative images taken at 8 h. Tube lengths were measured using ImageJ (version 1.47d). The experiment was performed three times, and the data are presented as the mean + SEM (right) (e). Ax, axitinib; CM, conditioned medium; IB, immunoblotting; Im, imatinib; IP, immunoprecipitation; VC, vehicle control; LO, Lipofectamine-only treated cells; Ut, untreated MLS cells. A paired, two-tailed *t*-test was performed. ^*∗*^ *p* < 0.05; ^*∗∗*^ *p* < 0.01; ^*∗∗∗*^ *p* < 0.001; ^*∗∗∗∗*^ *p* < 0.0001.

First, we assessed the effect of axitinib on phosphorylation, and thereby activation, of its angiogenesis- and cell-proliferation-promoting RTK targets (VEGFR1/2/3, platelet-derived growth factor receptor *α*/*β* (PDGFR*α*/*β*) and c-Kit). Using a MILLIPLEX assay for phosphorylated c-Kit, PDGFR*α*, and PDGFR*β*, we found a significant reduction in phospho-c-Kit (MLS 402, *p* = 0.0122; MLS 1765, *p* = 0.0016) and phospho-PDGFR*β* (MLS 402 and MLS 1765, *p* < 0.0001) following axitinib treatment, compared to controls (Figure 4(a)). There was also a significant reduction in phospho-PDGFR*β* (MLS 402 and MLS 1765, *p* = 0.0002) and phospho-c-Kit (MLS 402, *p* = 0.0026; MLS 1765, *p* = 0.0022) with imatinib treatment, compared with the vehicle control. Phospho-PDGFR*α* levels were below the level of detection in these cells. Moreover, MLS 1765 cells treated with axitinib had significantly reduced phosphorylated VEGFR3 compared to the vehicle control (*p* = 0.0128), although there was no change in the total levels of VEGFR3 (Figure 4(b)). These data indicate that axitinib inhibits the activation of the angiogenesis- and cell-proliferation-promoting receptors VEGFR3, c-Kit, and PDGFR*β*.

### 3.6. Axitinib Inhibits the Phosphorylation of Secondary Signaling Molecules

To determine the intracellular effects of axitinib treatment, we performed a Bio-Plex assay to examine the phosphorylation of intracellular signaling molecules that are known to be downstream of angiogenic receptors: AKT, ERK1/2, I*κ*B*α*, JNK1/2, and p38 MAPK. We found a significant reduction in the phosphorylation of AKT (MLS 402, *p* = 0.059; MLS 1765, *p* = 0.029) and a significant reduction in phosphorylation of ERK1/2 following axitinib treatment (MLS 402, *p* = 0.02; MLS 1765, *p* = 0.036) for both cell lines (Figure 4(c)). There was no change in the phosphorylation of I*κ*B*α*, JNK1/2, and p38 MAPK (data not shown). These data indicate that the effect of axitinib is likely mediated through a reduction in both ERK1/2 and AKT activity [25].

### 3.7. Axitinib Inhibits Angiogenic Properties

To ascertain whether axitinib also inhibits the expression of soluble angiogenic factors by myxoid liposarcoma cells, we examined VEGFR and VEGF expression. Axitinib treatment significantly decreased VEGFR1, VEGFR3, VEGFA, and VEGFB in MLS 402 cells (*p* = 0.0005, *p* = 0.0005, *p* < 0.0001, and *p* < 0.0001, resp.) and VEGFR1 and VEGFA in MLS 1765 cells (*p* = 0.0015 and *p* < 0.0001, resp.), compared with vehicle control (Figure 4(d)). Furthermore, in a tube formation assay, conditioned medium from MLS 402 cells that had been treated with axitinib induced significantly less tube formation than conditioned medium from vehicle-treated MLS 402 cells (*p* = 0.028) (Figure 4(e)).

### 3.8. Axitinib Halts Cell Cycle Progression and Induces Apoptosis

**Figure 5:**
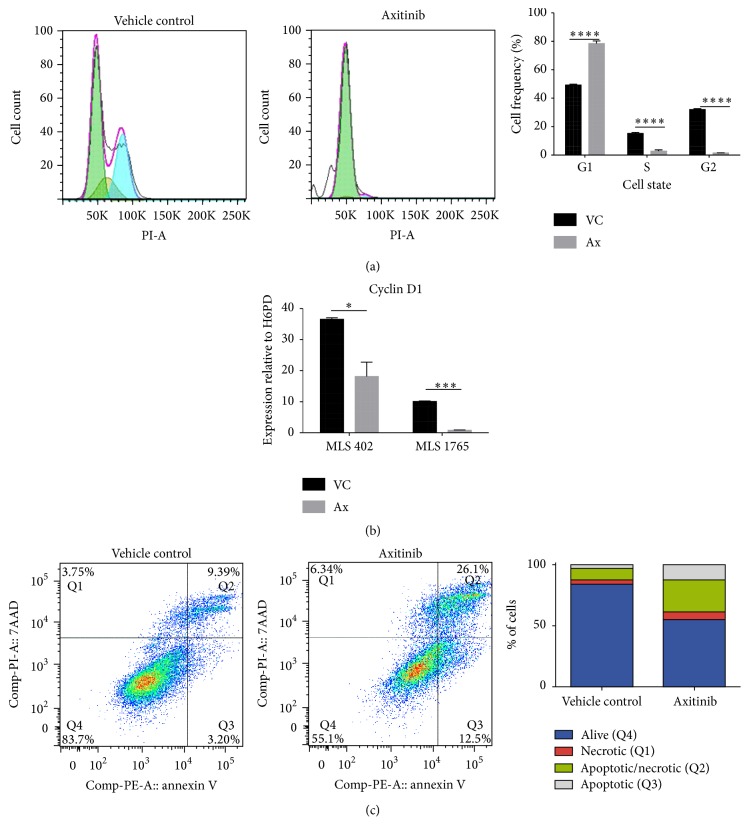
Axitinib affects cell cycle and apoptosis. MLS 1765 cells were treated with IC_50_ axitinib (3.2 *μ*M) or vehicle control overnight and then examined using a cell cycle assay. Green represents G1; yellow, S; and blue, G2. Three biological replicates were tested. The data are presented as the mean + SEM (a). MLS 402 and MLS 1765 cells were treated with IC_50_ axitinib (MLS 402, 1.2 *μ*M; MLS 1765, 3.2 *μ*M) or vehicle control overnight and then analyzed by RT-qPCR for the expression of cyclin D1 (b). MLS 1765 cells were treated overnight with IC_50_ axitinib or vehicle control and then assessed using an annexin V apoptosis assay. Biological triplicates were performed. Data represent an individual test that was representative of repeats (c). Ax, axitinib; VC, vehicle control; LO, Lipofectamine-only treated cells; Ut, untreated MLS cells. A paired, two-tailed *t*-test was performed. ^*∗*^ *p* < 0.05; ^*∗∗∗*^ *p* < 0.001; ^*∗∗∗∗*^ *p* < 0.0001.

To further characterize the antitumor effects of axitinib on myxoid liposarcoma cell lines, we performed cell cycle assays and annexin V apoptosis assays. Compared with vehicle, axitinib-treated MLS 1765 cell populations had a higher proportion of cells in the G1 phase of the cell cycle, a significant reduction in the proportion of cells in S phase, and almost no cells in G2 (*p* < 0.0001) (Figure 5(a)). This result clearly shows that axitinib treatment inhibits the progress of myxoid liposarcoma cells through the cell cycle. To verify this finding, we also examined the expression of cyclin D1, a key regulator of the cell cycle, which is required for G1/S transition. We observed a significant reduction in the expression of cyclin D1 in both cell lines (MLS 402, *p* = 0.0162; MLS 1765, *p* < 0.001) after axitinib treatment (Figure 5(b)). Reduced cell cycle progression and cyclin D1 expression would result in decreased cell proliferation.

To determine whether axitinib reduced cell survival via cell apoptosis and necrosis, we measured induction of the apoptotic marker annexin V. Axitinib treatment increased the proportion of cells that were in early apoptosis and were annexin V-positive (Q3) (MLS 1765 cells, vehicle, 3% positive; axitinib, 13% positive) (Figure 5(c)). Necrosis was also assessed, by measuring the necrosis marker 7AAD. Similarly, axitinib increased the proportion of 7AAD positive cells (Q1) (MLS 1765 cells, vehicle, 4% positive cells; axitinib, 6%). Cells that were positive for both annexin V and 7AAD (Q2), representing late apoptosis, were also increased following axitinib treatment (MLS 1765 cells, vehicle, 9% positive; axitinib 26% positive). Together, these data indicate that axitinib reduces cell proliferation and survival by inhibiting cell cycle progression and inducing cell apoptosis and necrosis.

### 3.9. Axitinib Inhibits Tumor Growth* In Vivo*

**Figure 6:**
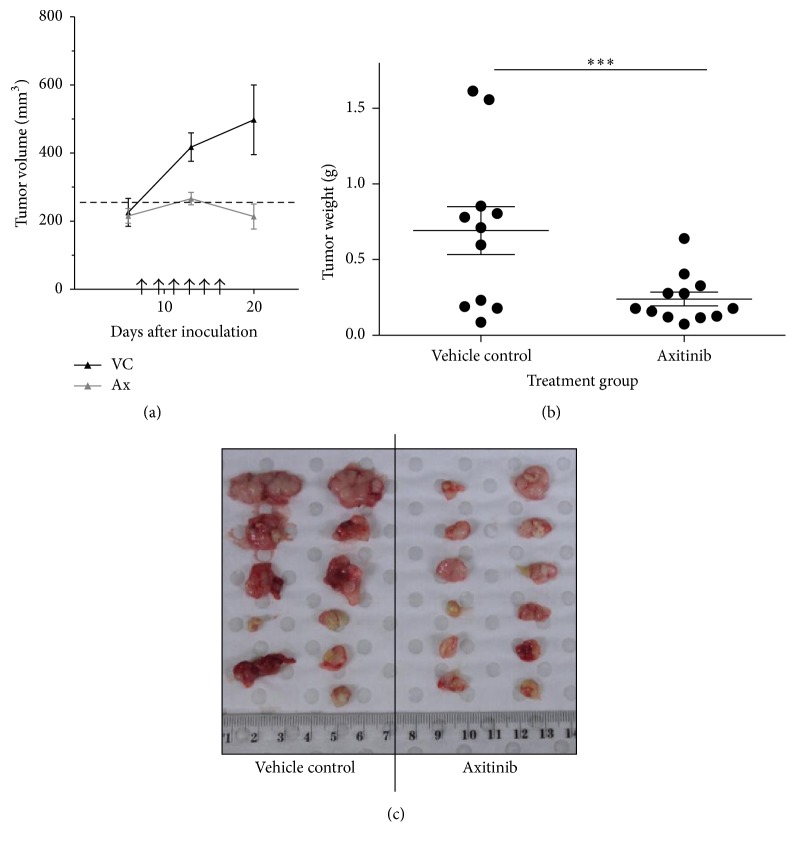
Axitinib inhibits tumor growth* in vivo*. Serially passaged (P5) MLS 1765 xenografts were transplanted into new NOD-SCID mice. When tumors reached approximately 200 mm^3^, mice were injected with 30 mg/kg axitinib or vehicle control (six mice per group) every second day for 12 days (day 7 to 18 after inoculation), as indicated by arrows and tumor size measured by digital calipers. Differences in tumor size were assessed by paired, two-tailed *t*-test. The data are presented as the mean ± SEM. The dashed line represents the average tumor size when treatment commenced (a). On day 20, mice were culled, and tumors were excised, weighed (b), and photographed (c). Differences in tumor weight were assessed by paired, two-tailed *t*-test. In (b), all individual data are presented, and horizontal lines indicate the mean ± SEM. In (c), all tumors are shown; note that one tumor did not grow.

To assess whether axitinib also has activity* in vivo*, we established an animal xenograft model using MLS 1765 cells. Treatment of xenografts commenced on day 7 after inoculation when the tumors were 100–200 mm^3^. By day 6 of treatment (day 13 after inoculation), the axitinib-treated tumors (266.3 ± 18.3 mm^3^) were significantly smaller than the vehicle controls (417.6 ± 41.6 mm^3^), as determined by caliper measurements. Axitinib-treated tumors were significantly smaller (213.8 ± 36.8 mm^3^) than vehicle-treated tumors (497.7 ± 102.3 mm^3^) by day 20 (*p* = 0.014) (Figure 6(a)). At the end of the experiment (day 20 after inoculation, because of ethical limits), tumors were measured, excised, weighed (Figure 6(b)), and photographed (Figure 6(c)). The observed variation in tumor size within groups was consistent with that observed by us and others in xenograft experiments. There was also a significant reduction in the weight of the axitinib-treated tumors (0.25 ± 0.05 g) compared with the vehicle controls (0.69 ± 0.16 g) (*p* = 0.0096). After four treatment doses, the tumors in the mice that received vehicle only had, on average, doubled in volume, whereas those in the treatment group had become static. By day 21, the vehicle-treated tumors had tripled in volume, whereas those in the treatment group remained static. Therefore, axitinib significantly reduced myxoid liposarcoma tumor growth* in vivo*.

## 4. Discussion

This study evaluated the molecular basis of tumorigenesis in myxoid liposarcoma and identified a number of potential therapeutics. Specifically, this study characterized the importance of VEGF receptors and ligands to myxoid liposarcoma cell survival and the efficacy of agents that target VEGF and VEGFR signaling, such as axitinib and 4EGI-1.

Myxoid liposarcoma is a rare malignancy that is characterized by the expression of the fusion protein FUS-CHOP [11]. The knockdown of FUS-CHOP in myxoid liposarcoma cells inhibited cell growth, induced cell cycle arrest, and reduced expression of VEGFR1 and the angiogenic ligand VEGFA. These findings suggest that FUS-CHOP mediates (at least in part) cell transforming activity by inducing an autocrine angiogenic signaling loop. This hypothesis is consistent with the previously reported 20-fold increase in VEGFR1 expression in cells expressing exogenous FUS-CHOP [23]. Furthermore, when the FUS-CHOP-negative cell line SW872 was treated with axitinib or sunitinib, the IC_50_ values were significantly elevated (6.2 *μ*M and 15.1 *μ*M, resp.) compared with the MLS FUS-CHOP-positive cell lines, indicating reduced sensitivity to VEGFR inhibition. These data indicate that the myxoid liposarcoma cell lines demonstrated increased sensitivity to the inhibition of angiogenic factors, indicating a possible mechanism of tumor growth.

Our finding that a VEGFR3 ligand trap, which binds VEGFC and VEGFD, markedly inhibited the growth of myxoid liposarcoma cells confirms that soluble angiogenic factors at least partly drive the growth of these cells. Avastin is a monoclonal antibody that targets VEGFA, the primary ligand responsible for angiogenesis. Although myxoid liposarcoma tumors express VEGFA, the expression of VEGFR2 (the primary receptor for VEGFA) is negligible in these cells. As the expression of VEGFR3 was elevated in the myxoid liposarcoma cell lines, it is possible that targeting VEGFR3 and/or the ligands VEGFC or VEGFD would be beneficial. As these reagents are not readily available in the clinic, targeting VEGFR receptors provides a more efficient option. In this way, targeting the angiogenic pathway in myxoid liposarcoma cells is still a viable option, whereas the utility of Avastin may be limited.

The overexpression of FUS-CHOP in several cell lines has resulted in the upregulation of* PDGFRA, HGF, MET, IL6* [8], and* VEGFR* genes [23]. From our drug analysis data (Supplementary Table S1), the two reagents that were most effective at reducing myxoid liposarcoma cell growth (i.e., the two with the lowest IC_50_ values) and that specifically targeted these RTKs were axitinib and sunitinib. Sorafenib, which targets VEGFR2/3, PDGFR*β*, and BRAF, exhibited high IC_50_s, indicating that the FUS-CHOP-containing cells were more dependent on VEGFR1 and PDGFR*α* signaling. This was, however, not the case for FUS-CHOP-negative cells, which exhibited a very low IC_50_ for sorafenib. These data indicate that FUS-CHOP has a regulatory role in RTK expression and results in differential responses to targeted therapeutics.

As can be seen, MLS-402 and MLS-1765 were significantly suppressed by axitinib and sunitinib, and owing to other growth-promoting genes, combining these drugs with other inhibitors further improved their efficacy. When FUS-CHOP was silenced in the two myxoid liposarcoma cells, up to 60% growth inhibition occurred. Furthermore, FUS-CHOP silencing also significantly reduced the expression of VEGFR1 (the cells expressed negligible VEGFR2) and prevented endothelial cell tube formation (an indicator of angiogenesis). Together, these data demonstrate that the contribution of FUS-CHOP expression to myxoid liposarcoma cell growth is significant and that targeting the fusion gene and/or its downstream targets such as VEGFR1 and PDGFR*α* induces significant inhibition of myxoid liposarcoma tumor growth.

Another protein known to promote angiogenesis is eIF4E. Others have shown that FUS-CHOP binds the promoter of the oncogenic transcription factor eIF4E, leading to its overexpression [6]. Our findings demonstrate that eIF4E expression is critical for myxoid liposarcoma cell survival and proliferation. Both chemical inhibition and siRNA knockdown of eIF4E markedly reduced the viability of two myxoid liposarcoma cell lines, demonstrating that the expression and activity of eIF4E are required for myxoid liposarcoma cell growth. Similar to our finding that FUS-CHOP promotes angiogenesis, we also demonstrated that eIF4E promotes the production of angiogenic factors in myxoid liposarcoma cell lines, as eIF4E siRNA knockdown significantly reduced the expression of VEGFA, VEGFB, and VEGFR3. Similarly, pharmacologic inhibition of eIF4E by 4EGI-1 decreased the expression of VEGFR1, VEGFR3, VEGFA, and VEGFB in myxoid liposarcoma cell lines. This finding suggests that eIF4E upregulates VEGFR signaling, thus contributing to myxoid liposarcoma tumorigenesis. Others have shown that overexpression of eIF4E can upregulate VEGFA expression [26, 27]. The previously demonstrated ability of VEGFA to increase VEGFR1 expression in HUVECs and developing endothelial cells [28, 29] may explain why we observed a decrease in VEGFA and VEGFR1 expression with eIF4E inhibition. Conditioned medium from eIF4E-directed siRNA-treated myxoid liposarcoma cells was less stimulatory in a tube formation assay than the vehicle control. This observation provides a functional demonstration of the link between the activity of eIF4E and the stimulation of angiogenesis. Furthermore, these studies confirm the importance of VEGFR signaling in myxoid liposarcoma cell lines.

The agent 4EGI-1 is a highly specific and competitive inhibitor of the interaction between eIF4E and eIF4G with *K*_*D*_ of 25 *μ*M and specifically inhibits cap-dependent translation through the upregulation of 4E-BP-1 [30]. The myxoid liposarcoma cells used in this study exhibited IC_50_ values of 8.2 and 4.8 *μ*M, which are below the *K*_*D*_ range indicating strong sensitivity to the agent. By contrast, the FUS-CHOP-negative liposarcoma line SW872 exhibited an IC_50_ of 25 *μ*M, indicating that these cells were not sensitive to the agent (Supplementary Table S1 and Supplementary Figure S11). Together, these findings indicate that the 4EGI-1 inhibitor was highly specific for eIF4E, with an increased sensitivity of the myxoid liposarcoma cells to eIF4E inhibition. Further evaluation of this agent for the clinic is therefore warranted for FUS-CHOP-positive tumors.

Our initial drug screen identified axitinib as a potent inhibitor of myxoid liposarcoma cell lines. Axitinib inhibited cell growth through targeting angiogenesis, which is an important process for the survival of myxoid liposarcoma cell lines and in the clinical progression of myxoid liposarcoma [19, 31, 32]. Our results revealed a significant decrease in the total levels of angiogenesis-associated molecules (VEGFR1, VEGFR3, VEGFA, and VEGFB), as well as decreased phosphorylation of VEGFR3, following axitinib treatment. We also demonstrated that unlike vehicle-pretreatment, conditioned medium from myxoid liposarcoma cells pretreated with axitinib had reduced expression of angiogenic ligands, as shown by reduced tube formation by endothelial cells. Angiogenesis is a critical process for tumorigenesis: inhibiting the activity of VEGFR1 or VEGFR3 reduces the proliferation of breast or colorectal cancer cell lines* in vitro* and inhibits tumor growth* in vivo* [33–36]. The importance of angiogenesis in our experiments is highlighted by the high sensitivity of the myxoid liposarcoma cell lines to agents that target the VEGFRs, such as axitinib, sorafenib, and sunitinib. Furthermore, the significant impact of the VEGFR1 and VEGFR3 ligand traps on cell survival establishes that VEGFR signaling may be critical for myxoid liposarcoma cell survival.

In addition to its antiangiogenic effects, axitinib limits the expression and/or activity of RTKs and downstream second messengers. Axitinib reduced the phosphorylation of VEGFR3, PDGFR*β*, and c-Kit, as well as that of the downstream molecules AKT (in MLS 1765) and ERK1/2. AKT and ERK1/2 are well-characterized mediators of VEGFR3, PDGFR*β*, and c-Kit signaling and are known to have critical roles in the regulation of cell survival, proliferation, and angiogenesis [37–41]. Moreover, axitinib was highly efficacious against myxoid liposarcoma xenografts in mice and should be explored as a potential treatment in the clinic. Our findings are further supported by Dossi et al., who demonstrated that the anticancer drug trabectedin inhibited the growth of myxoid liposarcoma xenografts by targeting angiogenesis [19].

Two soft-tissue sarcoma clinical trials have been performed using the targeted therapeutics pazopanib and sorafenib, with limited success [42, 43]. This lack of efficacy may reflect the specificity of these agents for VEGFRs [44], which is notably lower than that of axitinib. Furthermore, liposarcoma subtypes were not reported; therefore, these agents may not have been assessed against myxoid liposarcoma.

RTK inhibitors are highly effective when tumors are driven by a limited number of oncogenic promoters, such as BCR-ABL in CML. To overcome heterogeneous tumors such as myxoid liposarcoma tumors (and others), however, RTK inhibitors must target more than one oncogenic pathway (as axitinib does) or must be combined with other RTK inhibitors or chemotherapeutics. The combination of axitinib with doxorubicin [0.02 *μ*M] on MLS 1765 cells reduced the axitinib IC_50_ from 3.17 *μ*M to 0.5 *μ*M* in vitro*. It is therefore possible that this combination will be efficacious and will be less toxic than either agent used alone, and further investigation into axitinib's potential use in the clinic is warranted.

A significant finding from our studies is the importance of VEGFR3 in myxoid liposarcoma cell lines. Axitinib treatment significantly decreased the phosphorylation of VEGFR3, c-Kit, and PDGFR*β*. Moreover imatinib, which targets the same receptors as axitinib, except the VEGFRs, reduced the phosphorylation of c-Kit and PDGFR*β* but did not inhibit myxoid liposarcoma cell proliferation/survival at a biologically relevant dose. Therefore, the selective effectiveness of axitinib is likely due to axitinib's inhibition of VEGFRs. We have demonstrated that MLS 402 and MLS 1765 do not express VEGFR2, and we were unable to detect VEGFR1 phosphorylation in either of the two cell lines (data not shown), suggesting that VEGFR1 may not be active in myxoid liposarcoma cells. Importantly, the VEGFR3 ligand trap potently inhibited myxoid liposarcoma cell viability, whereas the VEGFR1 ligand trap had only modest effects. These results are supported by the finding that VEGFR tyrosine kinase inhibitor II, which targets VEGFR1, VEGFR2, c-Kit, and Src, at physiologically relevant doses did not significantly affect the proliferation of 3 myxoid liposarcoma cell lines (including MLS 402 and MLS 1765) or a fibrosarcoma cell line transfected with FUS-CHOP [23]. Therefore, VEGFR3 (and not VEGFR1 or VEGFR2) is the vital receptor for cell survival and proliferation in myxoid liposarcoma cells. Moreover, VEGFR3 has been shown to be important in other tumor types. Knockdown of VEGFR3 expression with siRNA in colorectal or breast cancer cell lines has been shown to reduce tumor cell proliferation* in vitro* and to significantly inhibit tumor growth* in vivo* [35, 36], indicating that VEGFR3 inhibition is sufficient to inhibit tumor growth. Collectively, our results highlight that VEGFR3 is a critical receptor for myxoid liposarcoma cell survival and suggest that myxoid liposarcoma cells may display oncogenic addiction to VEGFR3 signaling.

Myxoid liposarcoma is heterogeneous tumor and, as such, targeting FUS-CHOP or downstream targets of FUS-CHOP is only partially efficacious. By combining targeted therapeutics such as axitinib (which targets the downstream effectors of FUS-CHOP) with a chemotherapeutic (doxorubicin), an antibiotic (salinomycin), an apoptosis inducer (ABT-737), or the eIF4E inhibitor (4EGI-1), synergistic and antagonistic combinations were identified. Many of the combinations resulted in differential responses between the two cell lines, including antagonism in one cell line and synergism in the other (Supplementary Table S2). This indicated that any combination therapy undertaken in the future for myxoid liposarcoma will require personalization.

There are some caveats to our data that should be considered. Both of the cell lines used were immortalized with SV40, which may affect their biology in unexpected ways. In addition, whereas MLS 402 has a typical type 1 FUS-CHOP transcript found in 24% of patients with myxoid liposarcoma [45], MLS 1765 has a rare type 8 transcript [46]. Although both cell lines showed similar responses to the drugs evaluated* in vitro*, only the MLS 1765 cells grew as tumor xenografts in mice. Therefore, our* in vivo* data may not be representative of myxoid liposarcoma cell lines with more typical FUS-CHOP fusions. However, at the time of writing, these two cell lines were the only myxoid liposarcoma lines reported in the literature and as such were the best available cell models.

Three of the 11 compounds identified in our high-dose (10 *μ*M) initial screen have significant antiangiogenic activity. Titration of axitinib (Suppl. Table 1) showed its IC_50_ to be 1–3 *μ*M across the two myxoid liposarcoma cell lines. At this dose of axitinib, it is possible that some of its antitumor activity is mediated through its inhibition of RTKs such as PDGFR. We attempted to address this possibility in two ways. First, we used VEGFR1 and VEGFR3 ligand traps that are highly specific for angiogenic factors and have no off-target activity and found that these also inhibited the growth of both myxoid liposarcoma cell lines. Second, we assessed responses to imatinib, which targets the same kinases as axitinib except for the VEGFR family, and found that it had no antitumor activity against the myxoid liposarcoma cell lines. We did not have sufficient quantities of the ligand traps for the xenograft studies, which were therefore limited to axitinib. Consequently, we cannot conclusively state that the antitumor activity observed* in vivo* was purely mediated by axitinib's antiangiogenic activity, as some of it may have resulted from inhibition of other RTKs.

## 5. Conclusions

We found that VEGF receptor signaling, particularly through VEGFR3, has some role in the survival of myxoid liposarcoma cell lines. Furthermore, axitinib, a therapeutic agent that targets VEGFRs (including VEGFR3), shows antitumor activity against myxoid liposarcoma cell lines and significantly reduces the growth of MLS 1765 xenografts in mice. Our data suggest that axitinib should continue to be evaluated as a potential treatment for patients with myxoid liposarcoma.

## Supplementary Material

The Supplementary Material files contains the following: Supplementary Methods. Supplementary Table 1: Drug IC50 tabulation. Supplementary Table 2: Drug combination tabulation. Supplementary Figure S1: Drug dilution series to determine IC50: MLS 402 and MLS 1765. Supplementary Figure S2: Axitinib and salinomycin combination trials. Supplementary Figure S3: Axitinib and 4EGI-1 combination trials. Supplementary Figure S4: Salinomycin and 4EGI-1 combination trials. Supplementary Figure S5: Salinomycin and ABT-737 combination trials. Supplementary Figure S6: Trial of combinations of salinomycin/dasatinib and salinomycin/doxorubicin against MLS 1765. Supplementary Figure S7: Trial of combinations of ABT-737/4EGI-1 and ABT-737/axitinib against MLS 1765. Supplementary Figure S8: Trial of combinations of doxorubicin/4EGI-1 and doxorubicin/axitinib against MLS 1765. Supplementary Figure S9: Molecular characterization of MLS 402 cells. Supplementary Figure S10: Hematoxylin and eosin microphotographs of MLS 1765 xenograft tissue. Supplementary Figure S11: Drug dilution series to determine IC50: SW 872.
