# Supplementary material for: Axitinib Has Antiangiogenic and Antitumorigenic Activity in Myxoid Liposarcoma
Source: Sarcoma. 2016 Oct 16;2016:3484673. doi: 10.1155/2016/3484673 (PMC5086398; doi:10.1155/2016/3484673)
Supplement: Supplementary file 1 — The Supplementary Material files contains the following: Supplementary Methods. Supplementary Table 1: Drug IC50 tabulation. Supplementary Table 2: Drug combination tabulation. Supplementary Figure S1: Drug dilution series to determine IC50: MLS 402 and MLS 1765. Supplementary Figure S2: Axitinib and salinomycin combination trials. Supplementary Figure S3: Axitinib and 4EGI-1 combination trials. Supplementary Figure S4: Salinomycin and 4EGI-1 combination trials. Supplementary Figure S5: Salinomycin and ABT-737 combination trials. Supplementary Figure S6: Trial of combinations of salinomycin/dasatinib and salinomycin/doxorubicin against MLS 1765. Supplementary Figure S7: Trial of combinations of ABT-737/4EGI-1 and ABT-737/axitinib against MLS 1765. Supplementary Figure S8: Trial of combinations of doxorubicin/4EGI-1 and doxorubicin/axitinib against MLS 1765. Supplementary Figure S9: Molecular characterization of MLS 402 cells. Supplementary Figure S10: Hematoxylin and eosin microphotographs of MLS 1765 xenograft tissue. Supplementary Figure S11: Drug dilution series to determine IC50: SW 872. [file 3484673.f1.pdf]

SUPPLEMENTARY METHODS

Dose–response curves

Dose–response curves were determined as for the drug screen, except that a twofold dilution series of each drug was generated and then transferred to the plates. After incubation, an MTS assay was performed and the IC_{50} values for the drugs were calculated.

Drug combination trials

To determine the effect of combinations of two drugs, drug concentrations ranging from 4× IC_{50} to 1/4× IC_{50} were assayed alone and in combination in a checkerboard dilution series. The combination plates were prepared with pairs of agents arranged on each 96-well plate as a duplicate 6×6 matrix. This arrangement allowed the same data set to be interpreted using the Bliss independence, Loewe additivity, and combination index (CI) approaches [47].

Myxoid liposarcoma cells (1,000-2,000 cells per well) were incubated for 18 h at 37°C in 5% CO_2. A Zephyr liquid handling robot (Perkin Elmer, Waltham, MA) was used to create drug combinations and transfer them to

the plates, which were then incubated for 5 days at 37°C in 5% CO_2. An MTS assay was then performed.

The data were analyzed with several mathematical models to generate dose–response curves that show changes in absorbance with increasing drug concentrations. An isobologram that displays an additive effect of a drug combination (i.e., the effect is equal to that of each single agent added together) is depicted by points along the line of additivity. A synergistic effect is one that is greater than the combined effect of each single agent, which is shown by points below the line of additivity. An antagonistic effect is less than the additive effect and is denoted by points above the line of additivity. The CI plot depicts how many points are additive or synergistic, as shown by those points <1.0. The Chou curve demonstrates how well the data fit the CI plot: R^2 values >0.9 indicate that the CI plot is a reasonable representation of the combined effect of the two drugs.

47. Goldoni M, Johansson C. A mathematical approach to study combined effects of toxicants in vitro: evaluation of the Bliss independence criterion and the Loewe additivity model. Toxicology In Vitro. 2007;21:759-69.
