# Supplementary material for: Axitinib Has Antiangiogenic and Antitumorigenic Activity in Myxoid Liposarcoma
Source: Sarcoma. 2016 Oct 16;2016:3484673. doi: 10.1155/2016/3484673 (PMC5086398; doi:10.1155/2016/3484673)
Supplement: Supplementary file 2 [file 3484673.f2.pdf]

Drug	Target/activity	IC_{50} (µM) (MLS 402)	IC_{50} (µM) (MLS1765)	IC_{50} (µM) (SW872)
4EGI-1	eIF4E	8.20	4.80	24.80
ABT-737	BCL-2, BCL-W, BCL-X_L	14.12	12.83	ND
ABT-737 enantiomer	Mirror image of ABT-737	37.84	36.37	ND
Axitinib	C-Kit, PDGFRα/β, VEGFR1/2/3	1.20	3.17	6.20
Bortezomib*	proteasome	0.03	0.06	ND
CYT387	Jak1/Jak2	1.22	1.39	ND
Dasatinib	BCR-ABL, Src	1.57	4.00	ND
Docetaxel*	Anti-mitotic chemotherspeutic	1.02	ND	ND
Doxorubicin *	Anthracycline antibiotic	0.16	0.07	0.11
Floxuridine*	antimetabolite	0.02	0.04	ND
Imatinib	PDGFRα/β, c-Kit, BCR-ABL	22.66	35.67	39.10
Salinomycin	Potassium channel ionophore	1.34	1.29	0.82
Sorefenib	VEGFR2/3, PDGFRβ, BRAF	10.37	9.91	0.07
Sunitinib	VEGFR1/2/3, PDGFRα/β, c-Kit	3.80	1.70	15.10

Supplementary Table 1: Drug IC_{50} tabulation.

IC_{50} as determined using a dilution series of drugs that reduced MLS 402 and MLS 1765 and SW872 cell viability. The agents were plated in duplicate, and the assay was repeated twice or three times.

ND: not determined

*   chemotherapeutic

Treatment Combination	MLS 402	MLS 1765
Axitinib + salinomycin	A	S
Axitinib + ABT-737	ND	S
Salinomycin + ABT-737	S	S
Salinomycin + 4EGI-1	S	A
Axitinib + doxorubicin	ND	A
Doxorubicin + 4EGI-1	ND	A
ABT-737 + 4EGI-1	ND	A
Doxorubicin + salinomycin	ND	C
Axitinib + 4EGI-1	C	C

Supplementary Table 2: Drug combination tabulation.
This table lists the drug combinations investigated and identifies those that were additive (A), synergistic (S) or competitive/antagonistic (C). ND refers to not determined.

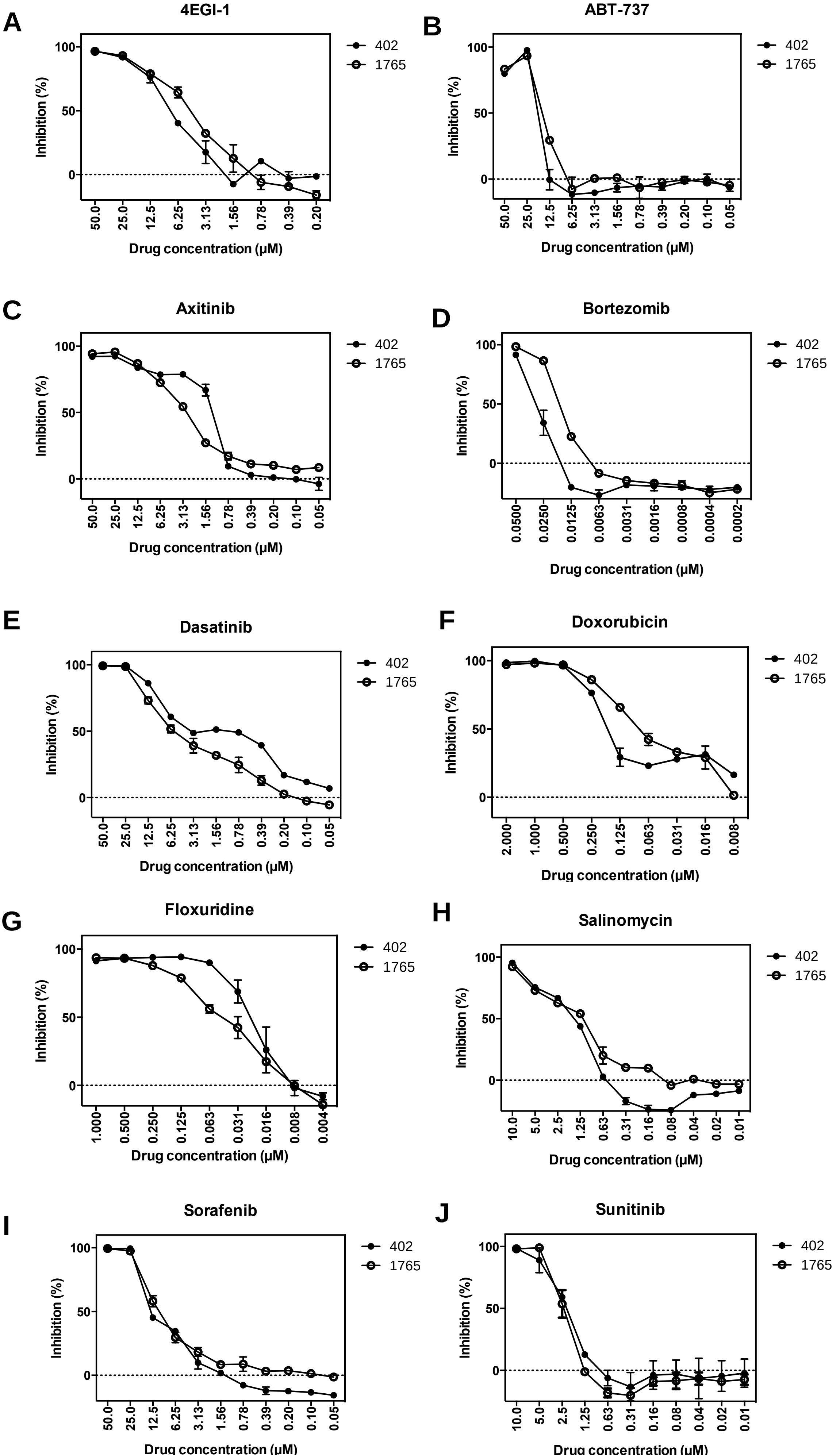

SUPPLEMENTARY FIGURE S1

Supplementary Figure S1: Drug dilution series to determine IC50
IC50 was determined using a dilution of drugs that inhibited MLS 402 and MLS 1765 cell viability. Technical and biological duplicates were performed. Graph points are mean ± SEM.

The combination of Axitinib and Salinomycin

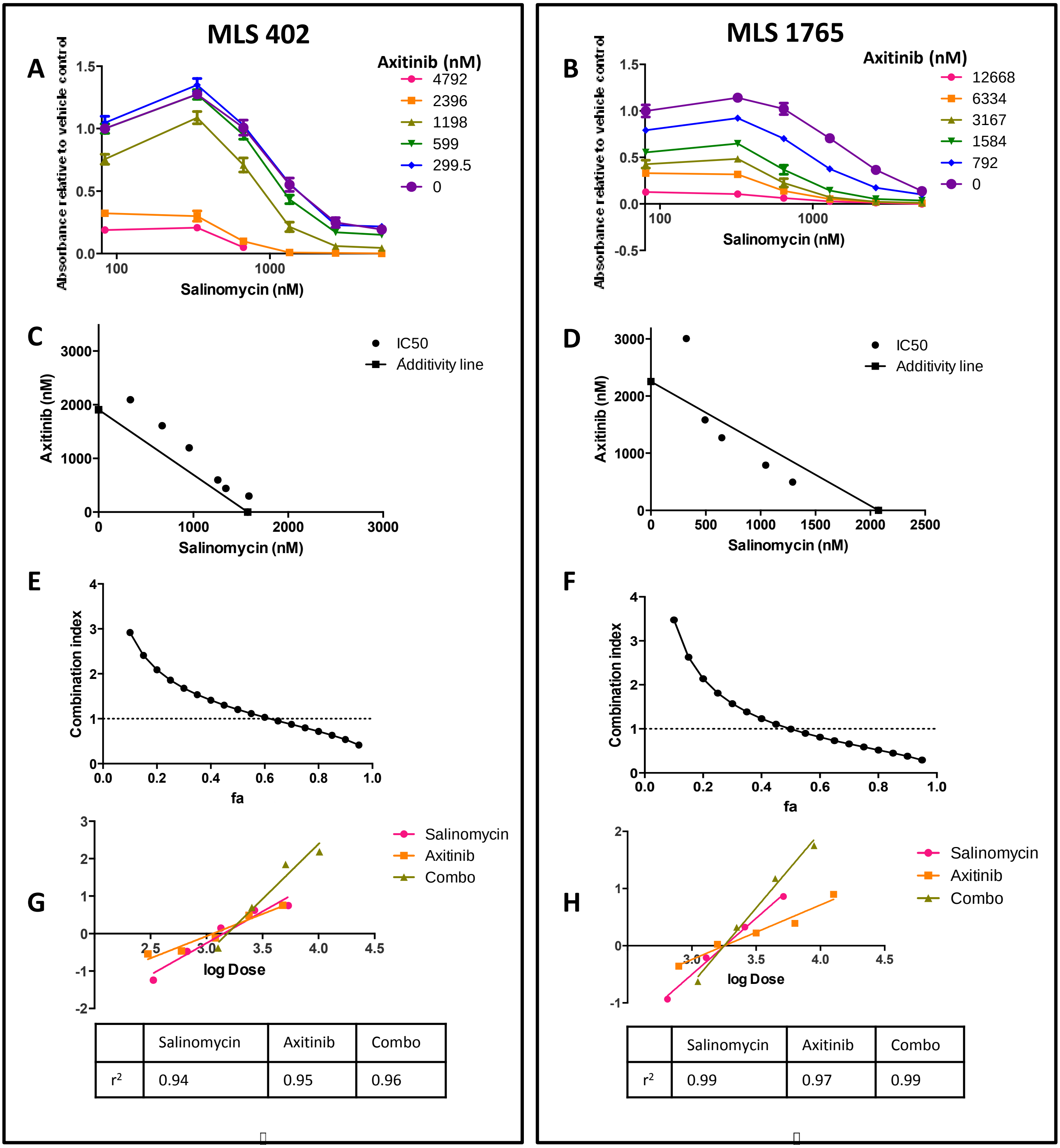

Supplementary Figure S2: Axitinib and salinomycin combination trials (A-H) The relationship between the drug combination of axitinib and salinomycin against MLS cell lines was analysed. MLS 402 and MLS 1765 cells were exposed to differing concentrations of two drugs for five days followed by MTS dye uptake. The dose response curves (A-B) demonstrated decreased viability indicator (absorbance) with increased drug concentrations. The isobolograms (C-D) indicated the relationship between the two drugs to be synergistic for MLS 1765 and additive for MLS 402. The Cl plots (E-F) showed the number of points that were additive or synergistic (below 1). The Chou curves (G-H) represent the mean effect plot, which shows how accurately the Cl plot depicted the data, above 0.9 is well represented.

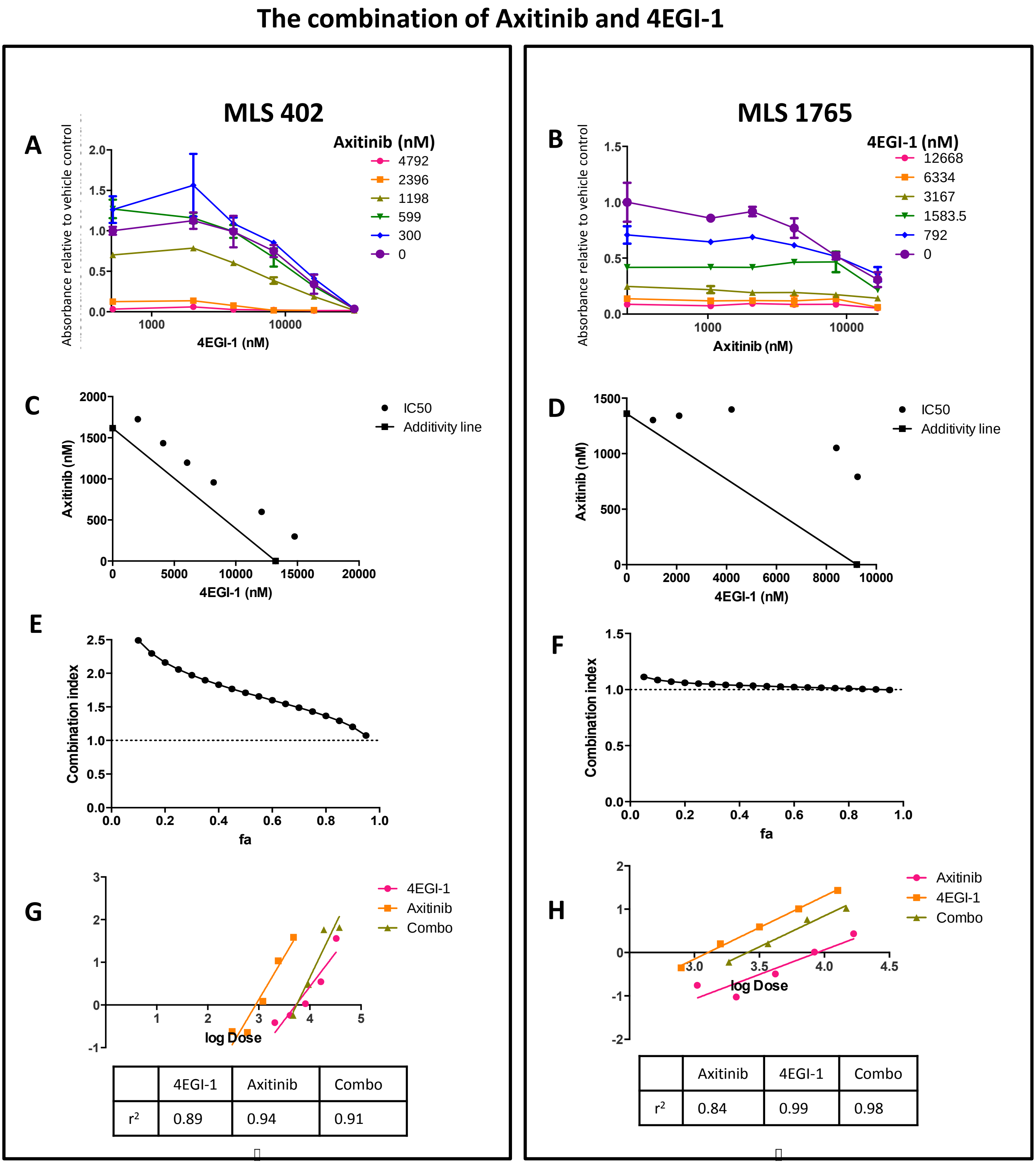

	4EGI-1	Axitinib	Combo
r^2	0.89	0.94	0.91

	Axitinib	4EGI-1	Combo
r^2	0.84	0.99	0.98

Supplementary Figure S3: Axitinib and 4EGI-1 combination trials. (A-H) The relationship between the drug combination of axitinib and 4EGI-1 against MLS cell lines was analysed. MLS 402 and MLS 1765 cells were exposed to differing concentrations of two drugs for five days as described in Supp Fig 1. The dose response curves (A-B) demonstrated decreased viability with increased drug concentrations. The isobolograms (C-D) indicated the relationship between the two drugs to be antagonistic for MLS 402 and MLS 1765. The CI plots (E-F) showed the number of points that were additive or synergistic (below 1). The Chou curves (G-H) represent the mean effect plot, which shows how accurately the CI plot depicted the data, above 0.9 is well represented.

The combination of Salinomycin and 4EGI-1

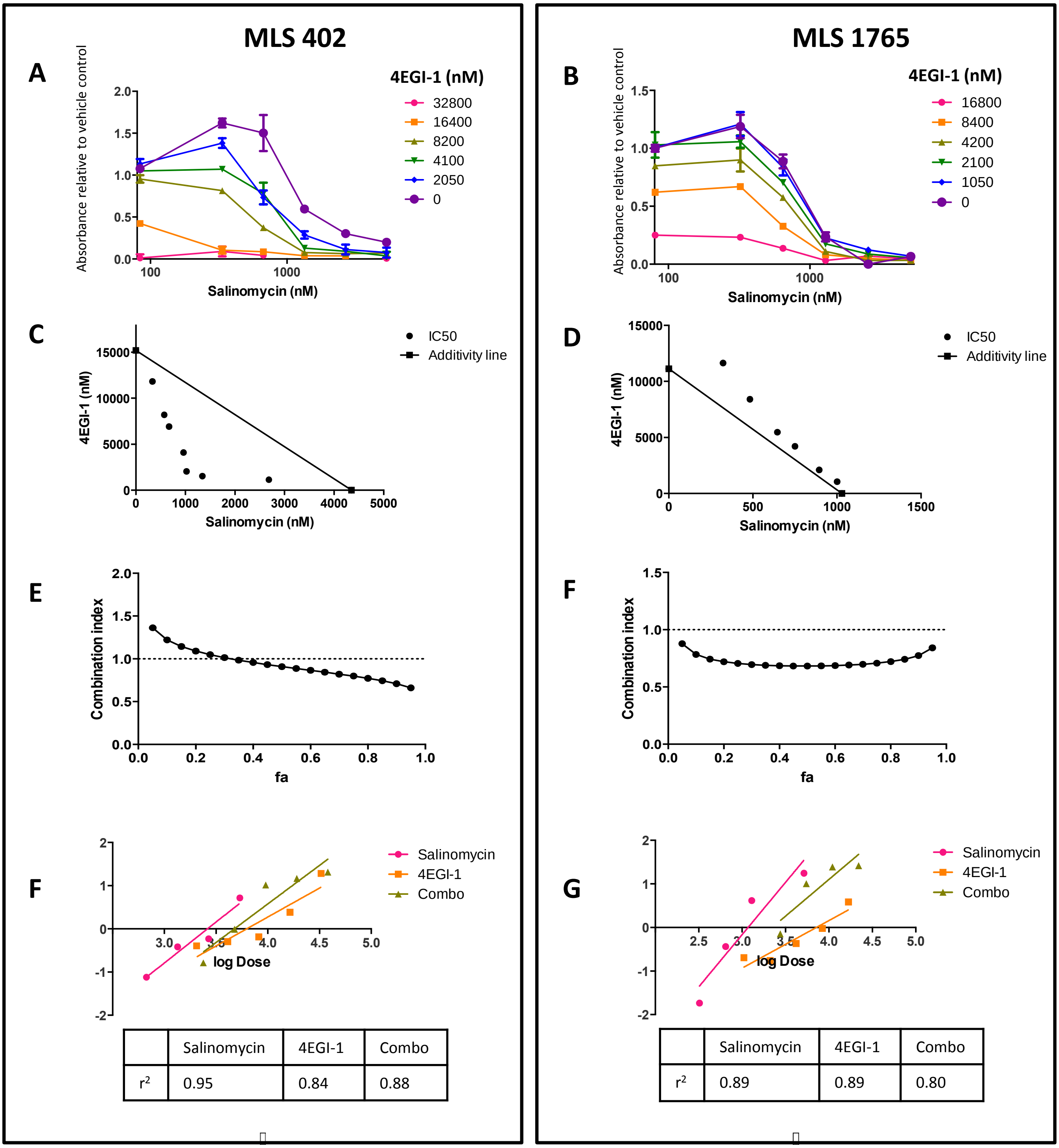

MLS 402:

	Salinomycin	4EGI-1	Combo
r^2	0.95	0.84	0.88

MLS 1765:

	Salinomycin	4EGI-1	Combo
r^2	0.89	0.89	0.80

Supplementary Figure S4: Salinomycin and 4EGI-1 combination trials. (A-H) The relationship between the drug combination of 4EGI-1 and salinomycin against MLS cell lines was analysed. MLS 402 and MLS 1765 cells were exposed to differing concentrations of two drugs for five days as described in Supp Fig 1. The dose response curves (A-B) demonstrated decreased viability with increased drug concentrations. The isobolograms (C-D) indicated the relationship between the two drugs to be synergistic for MLS 402 and additive for MLS 1765. The CI plots (G-H) showed the number of points that were additive or synergistic (below 1). The Chou curves (E-F) represent the mean effect plot, which shows how accurately the CI plot depicted the data, above 0.9 is well represented.

Combination of Salinomycin and ABT-737

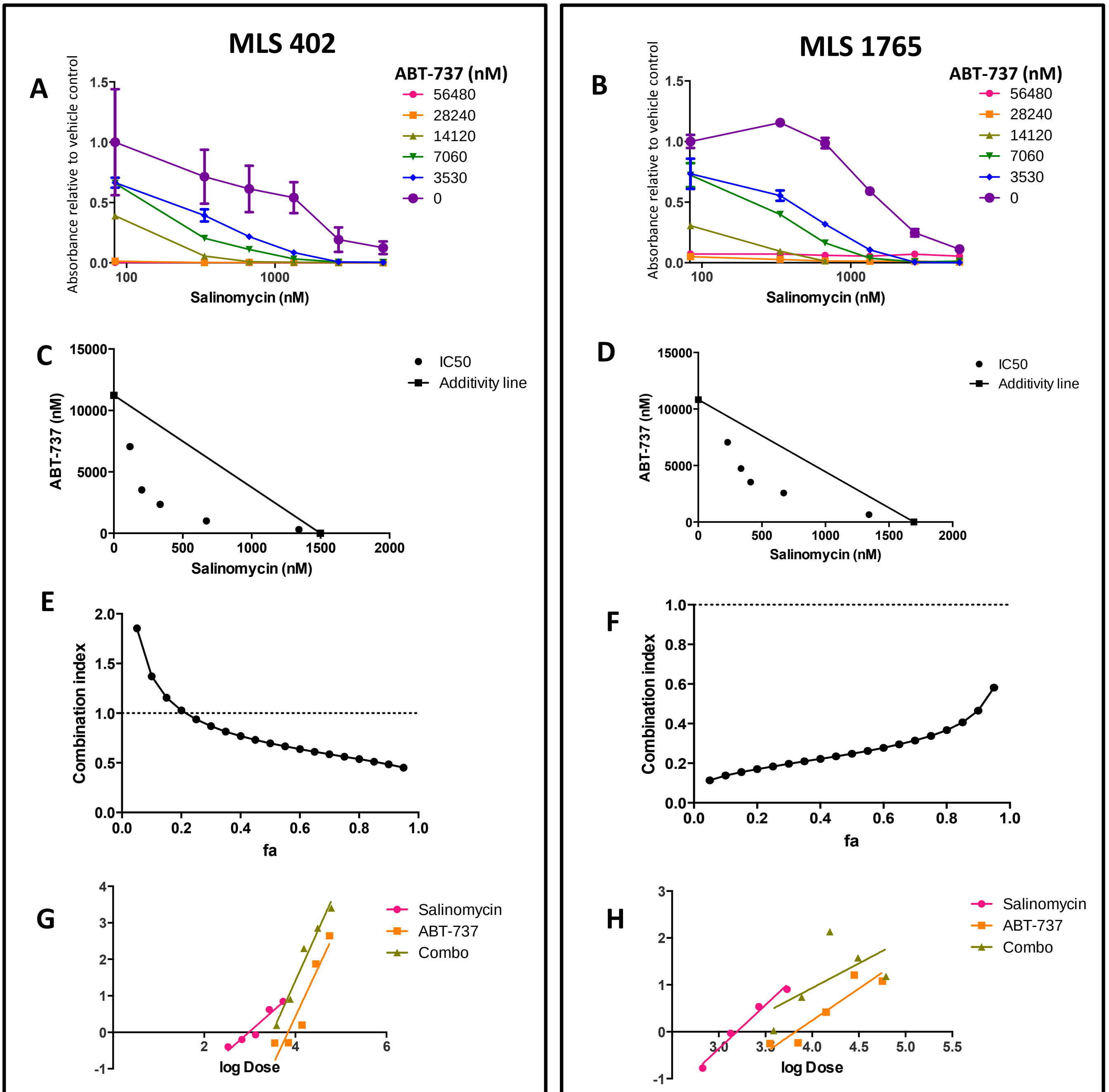

	Salinomycin	ABT-737	Combo
r^2	0.93	0.89	0.97

	Salinomycin	Axitinib	Combo
r^2	0.98	0.87	0.38

Supplementary Figure S5: Salinomyocin and ABT-737 combination trials. (A-H) The relationship between the drug combination of ABT-737 and salinomycin against MLS cell lines was examined. MLS 402 and MLS 1765 cells were exposed to differing concentrations of two drugs for five days as described in Supp Fig 1. The dose response curves (A-B) demonstrated decreased viability with increased drug concentrations. The isobolograms (C-D) indicated the relationship between the two drugs to be synergistic for both MLS cell lines. The Cl plots (G-H) showed the number of points that were additive or synergistic (below 1). The Chou curves (E-F) represent the mean effect plot, which shows how accurately the Cl plot depicted the data, above 0.9 is well represented.

The combination of Salinomycin with Dasatinib or Doxorubicin in MLS 1765

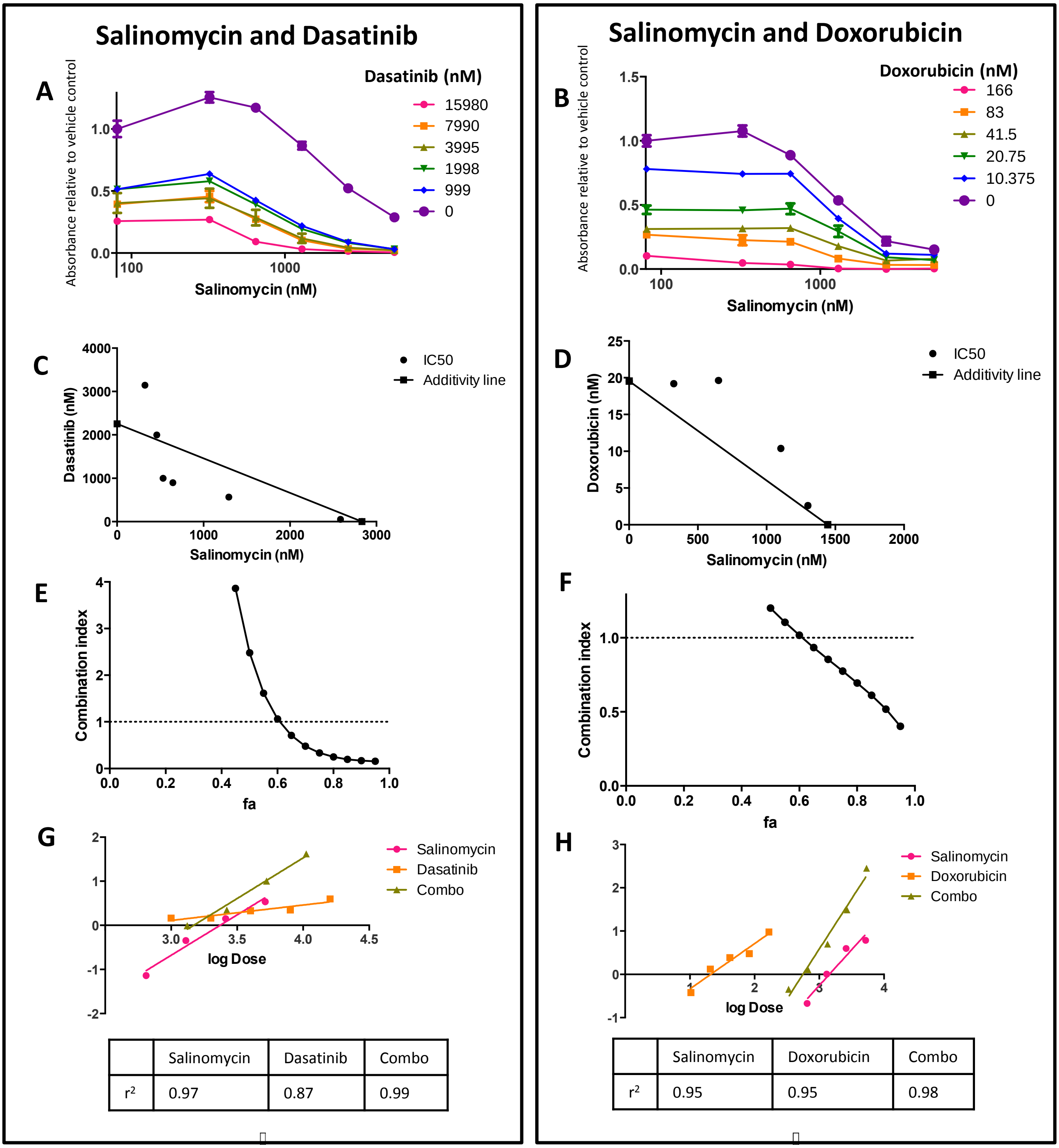

	Salinomycin	Dasatinib	Combo
r^2	0.97	0.87	0.99

	Salinomycin	Doxorubicin	Combo
r^2	0.95	0.95	0.98

Supplementary Figure S6: Trial of combinations of salinomycin/dasatinib and salinomycin/doxorubicin against MLS 1765. (A-H) The relationship between the drug combination of salinomycin and dasatinib or salinomycin and doxorubicin against an MLS cell line was examined. MLS 1765 cells were exposed to differing concentrations of two drugs for five days as described in Supp Fig 1. The dose response curve (A-B) demonstrated decreased viability with increased drug concentration. The isobologram (C-D) indicated the relationship between the two drugs to be synergistic for salinomycin and dasatinib but antagonistic for salinomycin and doxirubicin. The CI plot (E-F) showed the number of points that are additive or synergistic (below 1). The Chou curve (G-H) represents the mean effect plot, which showed how accurately the CI plot depicted the data, above 0.9 is well represented.

The combination of ABT-737 with 4EGI-1 or Axitinib in MLS 1765

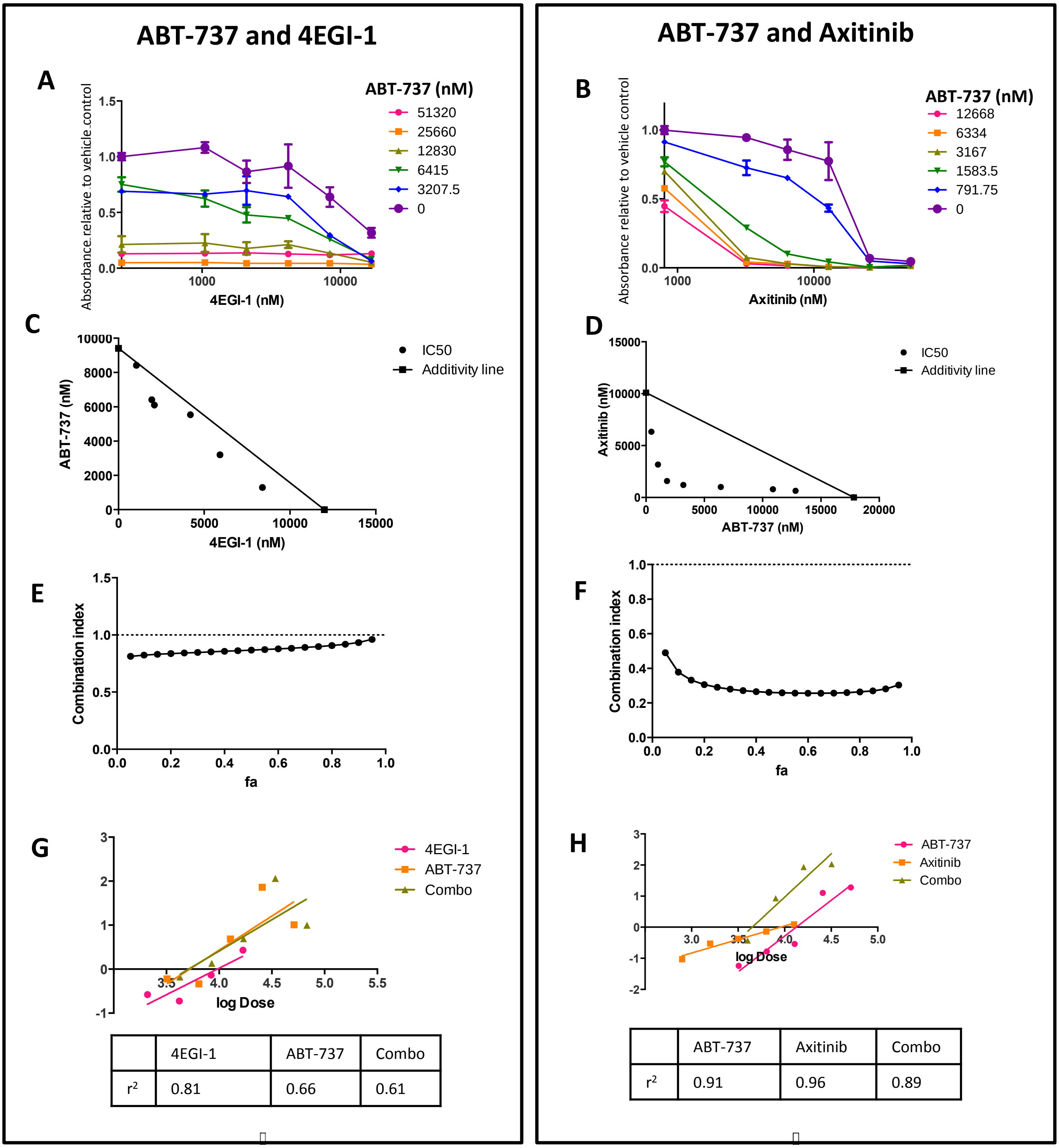

	4EGI-1	ABT-737	Combo
r^2	0.81	0.66	0.61

	ABT-737	Axitinib	Combo
r^2	0.91	0.96	0.89

Supplementary Figure S7: Trial of the combinations of ABT-737/4EGI-1 and ABT-737/axitinib against MLS 1765. The relationship between the drug combinations of 4EGI-1/ABT-737 and ABT-737/axitinib against an MLS cell line was examined. MLS 1765 cells were exposed to differing concentrations of two drugs for five days as described in Supp Fig 1. The dose response curves (A-B) demonstrated decreased viability with increased drug concentrations. The isobolograms (C-D) indicated the relationship between the two drugs to be more than additive for ABT-737/4EGI-1 and synergistic for ABT-737/axitinib drug combinations. The CI plots (E-F) showed the number of points that were additive or synergistic (below 1). The Chou curves (G-H) represent the mean effect plot, which showed how accurately the CI plot depicts the data, above 0.9 is well represented.

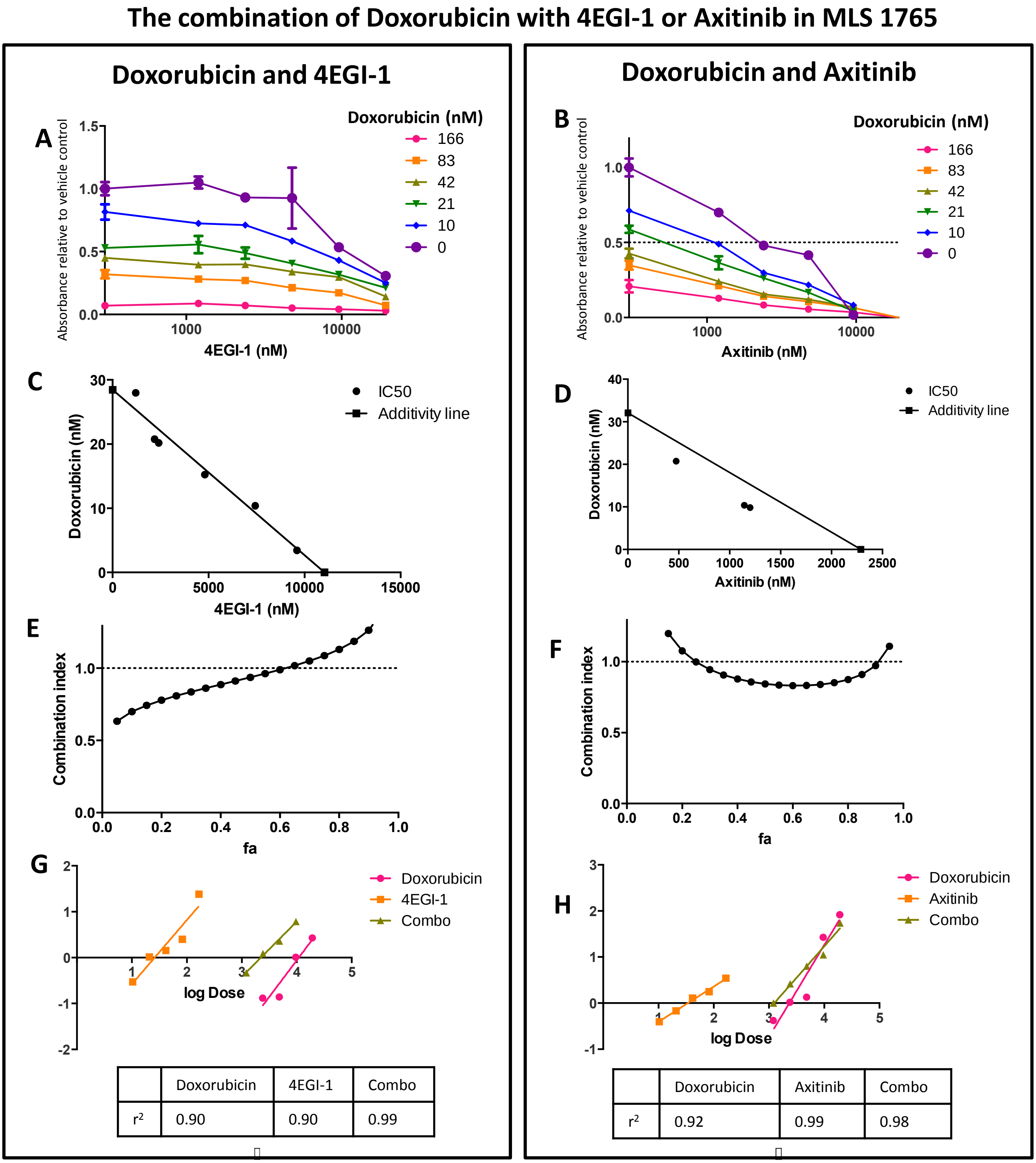

	Doxorubicin	4EGI-1	Combo
r^2	0.90	0.90	0.99

	Doxorubicin	Axitinib	Combo
r^2	0.92	0.99	0.98

Supplementary Figure S8: Trial of the combinations of doxorubicin/4EGI-1 and doxorubicin/axitinib against MLS 1765. (A-H)The relationship between the drug combinations of 4EGI-1/doxorubicin and axitinib/doxorubicin was examined. MLS 1765 were exposed to differing concentrations of two drugs for five days as described in Supp Fig 1. The dose response curves (A-B) demonstrated decreased viability with increased drug concentrations. The isobolograms (C-D) indicated the relationship between the two drugs to be additive for doxorubicin / 4EGI-1 and more than additive for doxorubicin/axitinib. The Cl plots (E-F) shows the number of points that were additive or synergistic (below 1). The Chou curves (G-H) represent the mean effect plot, which showed how accurately the Cl plot depicts the data, above 0.9 is well represented.

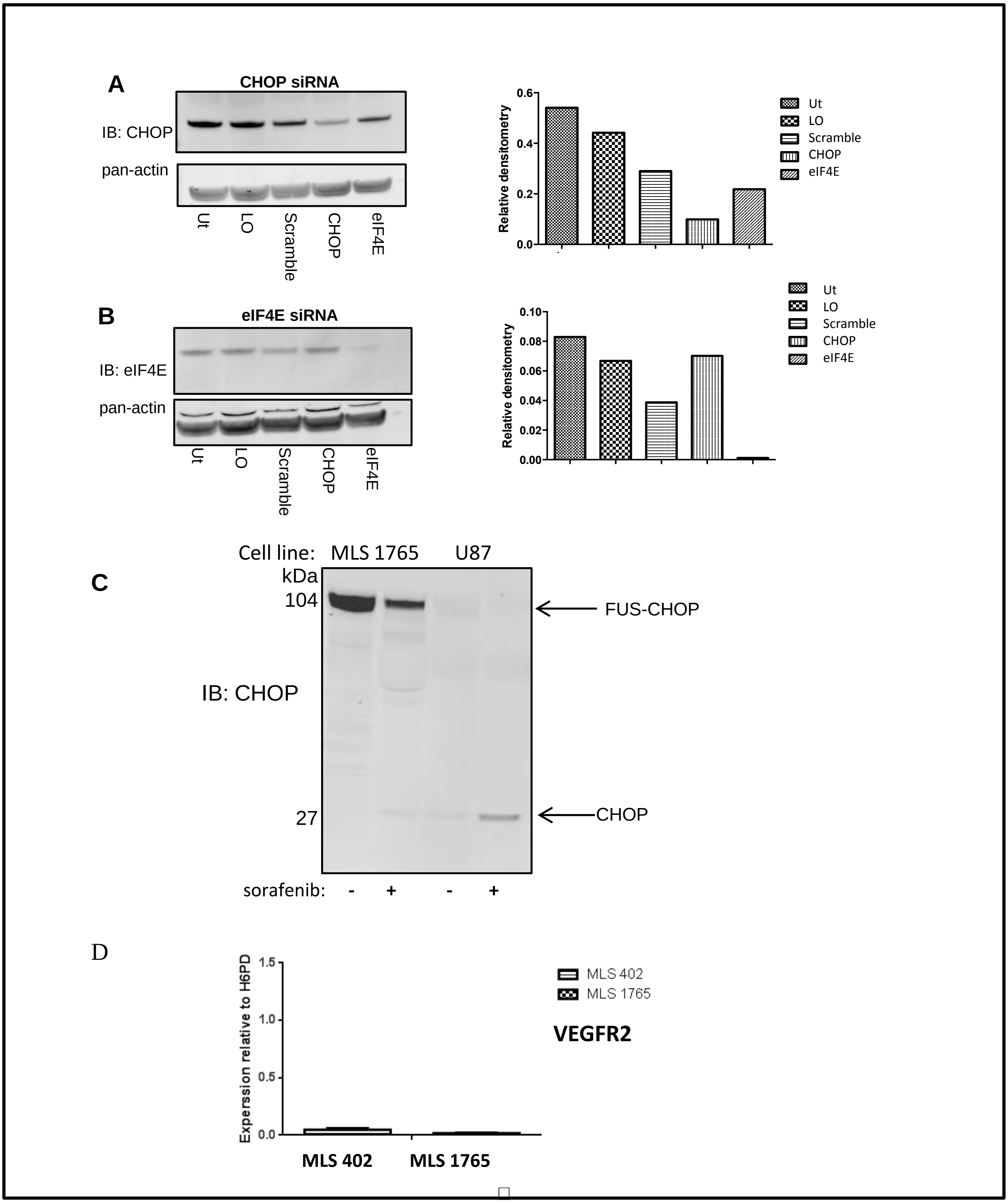

Supplementary Figure S9: Molecular characterization of MLS 402 cells: CHOP (A) and eIF4E (B) siRNA successfully reduced FUS-CHOP and eIF4E expression in MLS 402 cells by 60% and 75%, respectively. FUS-CHOP expression was demonstrated at 104 kDa (C); endogenous CHOP was not expressed in MLS 1765 but could be stimulated in U87 cells (a brain cancer cell line). VEGFR2 expression in MLS cell lines is negligible as determined by RT-qPCR on cDNA from MLS 402 and MLS 1765 cell lines (D). Bar graph shows mean +SEM.

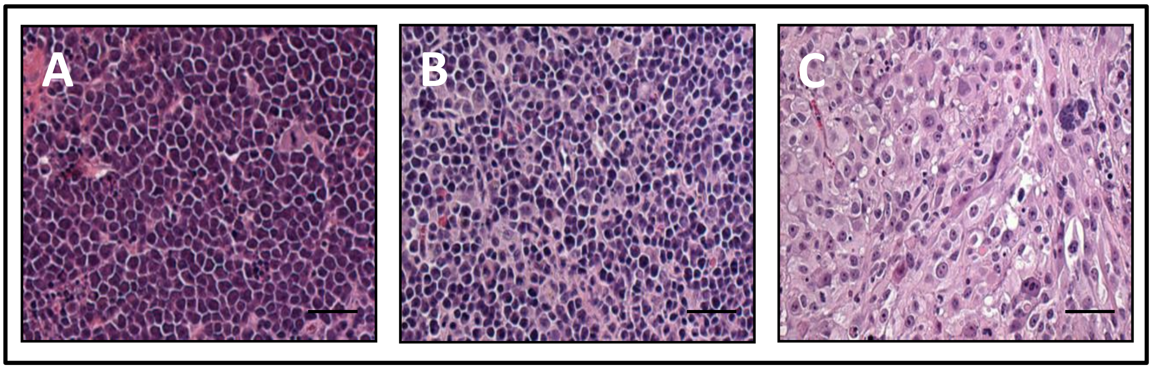

Supplementary Figure S10: Hematoxylin and eosin microphotographs of MLS 1765 xenograft tissue. MLS 1765 was serially passaged through NOD-SCID mice and collected at passage 1 (A), passage 2 (B) and passage 5 (C). The passage 5 sample was used for the therapeutic study. Scale bar, 50 µm.

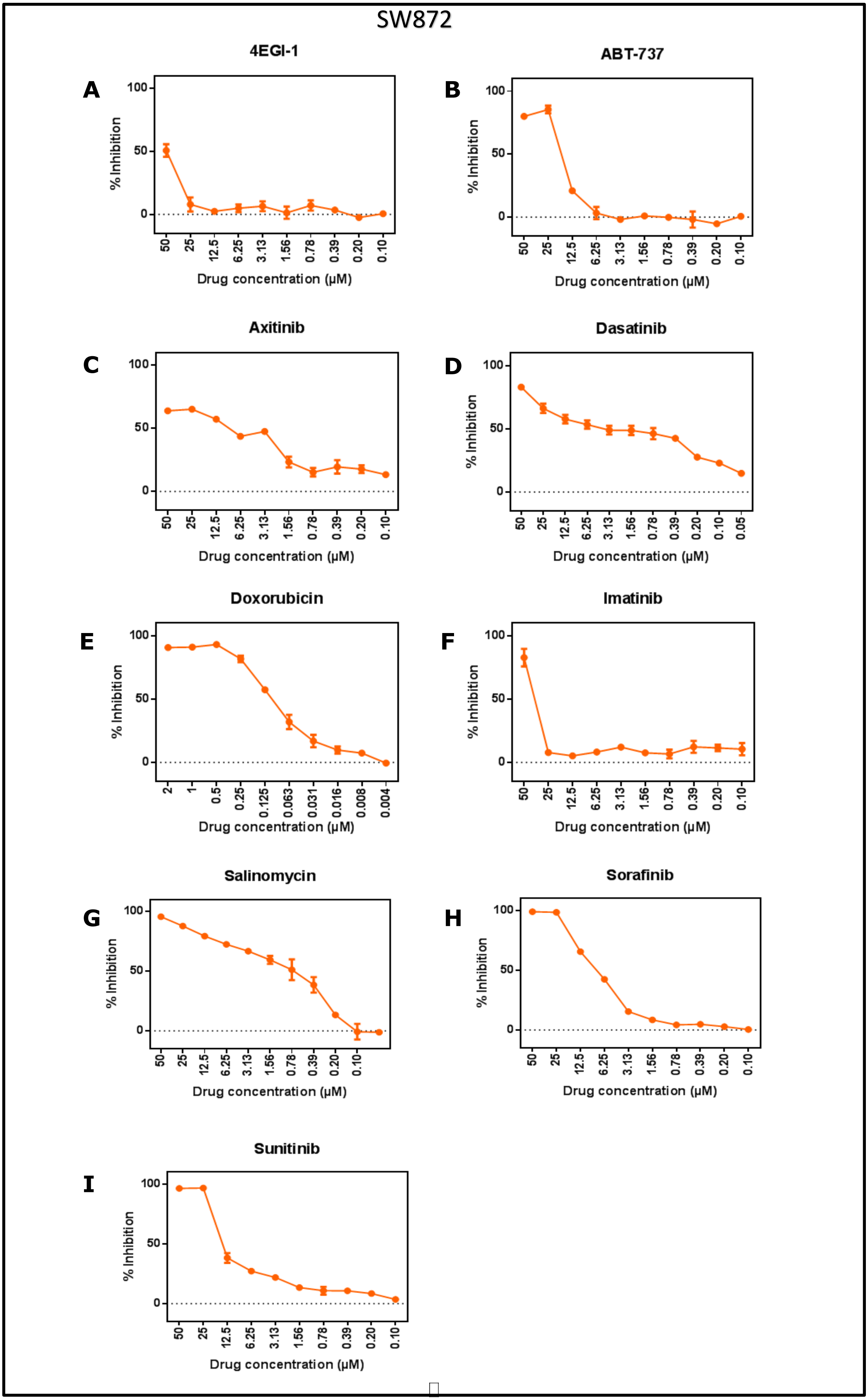

SUPPLEMENTARY FIGURE 11

Supplementary Figure S11: Drug dilution series to determine IC50. IC50 was determined using a dilution of drugs that inhibited SW872 cell viability. Technical and biological duplicates were performed.
